# Author Correction: Altered structural brain asymmetry in autism spectrum disorder in a study of 54 datasets

**DOI:** 10.1038/s41467-021-27519-7

**Published:** 2021-12-08

**Authors:** Merel C. Postema, Daan van Rooij, Evdokia Anagnostou, Celso Arango, Guillaume Auzias, Marlene Behrmann, Geraldo Busatto Filho, Sara Calderoni, Rosa Calvo, Eileen Daly, Christine Deruelle, Adriana Di Martino, Ilan Dinstein, Fabio Luis S. Duran, Sarah Durston, Christine Ecker, Stefan Ehrlich, Damien Fair, Jennifer Fedor, Xin Feng, Jackie Fitzgerald, Dorothea L. Floris, Christine M. Freitag, Louise Gallagher, David C. Glahn, Ilaria Gori, Shlomi Haar, Liesbeth Hoekstra, Neda Jahanshad, Maria Jalbrzikowski, Joost Janssen, Joseph A. King, Xiang Zhen Kong, Luisa Lazaro, Jason P. Lerch, Beatriz Luna, Mauricio M. Martinho, Jane McGrath, Sarah E. Medland, Filippo Muratori, Clodagh M. Murphy, Declan G. M. Murphy, Kirsten O’Hearn, Bob Oranje, Mara Parellada, Olga Puig, Alessandra Retico, Pedro Rosa, Katya Rubia, Devon Shook, Margot J. Taylor, Michela Tosetti, Gregory L. Wallace, Fengfeng Zhou, Paul M. Thompson, Simon E. Fisher, Jan K. Buitelaar, Clyde Francks

**Affiliations:** 1 Department of Language & Genetics, Max Planck Institute for Psycholinguistics, Nijmegen, The Netherlands; 2 Department of Cognitive Neuroscience, Donders Institute for Brain, Cognition and Behaviour, Donders Centre for Cognitive Neuroimaging, Radboud University Medical Centre, Nijmegen, The Netherlands; 3 Bloorview Research Institute, Holland Bloorview Kids Rehabilitation Hospital and Department of Pediatrics, University of Toronto, Toronto, ON USA; 4 Child and Adolescent Psychiatry Department, Gregorio Marañón General University Hospital, School of Medicine, Universidad Complutense, IiSGM, CIBERSAM, Madrid, Spain; 5 Institut de Neurosciences de la Timone, UMR 7289, Aix Marseille Université, CNRS, Marseille, France; 6 Department of Psychology, Carnegie Mellon University, Pittsburgh, PA USA; 7 Laboratory of Psychiatric Neuroimaging (LIM-21), Departamento e Instituto de Psiquiatria, Hospital das Clinicas HCFMUSP, Faculdade de Medicina, Universidade de Sao Paulo, Sao Paulo, SP Brazil; 8 IRCCS Stella Maris Foundation, viale del Tirreno 331, 56128 Pisa, Italy; 9 Department of Clinical and Experimental Medicine, University of Pisa, Pisa, Italy; 10 Department of Child and Adolescent Psychiatry and Psychology Hospital Clinic, Psychiatry Unit, Department of Medicine, 2017SGR881, University of Barcelona, IDIBAPS, CIBERSAM, Barcelona, Spain; 11 Department of Forensic and Neurodevelopmental Sciences, Institute of Psychiatry, Psychology & Neuroscience King’s College London, London, UK; 12 Institute for Pediatric Neuroscience, NYU Child Study Center, New York, NY USA; 13 Department of Psychology, Ben-Gurion University of the Negev, Beer Sheva, Israel; 14 Brain Center Rudolf Magnus, Department of Psychiatry, University Medical Center Utrecht, Utrecht, The Netherlands; 15 Department of Child and Adolescent Psychiatry, Psychosomatics and Psychotherapy, University Hospital, Goethe University Frankfurt am Main, Frankfurt, Germany; 16 Imaging Genetics Center, Mark and Mary Stevens Neuroimaging and Informatics Institute, Keck School of Medicine of USC, University of Southern California, Marina del Rey, CA USA; 17 Department of Child and Adolescent Psychiatry, Division of Psychological and Social Medicine and Developmental Neurosciences, Faculty of Medicine, TU Dresden, Dresden, Germany; 18 Department of Behavioral Neuroscience, Oregon Health & Science University, Portland, OR USA; 19 Department of Psychiatry, University of Pittsburgh, Pittsburgh, PA USA; 20 BioKnow Health Informatics Lab, College of Computer Science and Technology, and Key Laboratory of Symbolic Computation and Knowledge Engineering of Ministry of Education, Jilin University, Changchun, Jilin 130012 China; 21 Department of Psychiatry, School of Medicine, Trinity College, Dublin, Ireland; 22 The Trinity College Institute of Neuroscience, Trinity College, Dublin, Ireland; 23 Department of Psychiatry, Boston Children’s Hospital and Harvard Medical School, Boston, MA 02115-5724 USA; 24 Olin Neuropsychiatric Research Center, Hartford, CT USA; 25 National Institute for Nuclear Physics, Pisa Division, Largo B. Pontecorvo 3, 56124 Pisa, Italy; 26 Department of Bioengineering, Imperial College London, London, UK; 27 Karakter Child and Adolescent Psychiatry University Centre, Nijmegen, The Netherlands; 28 Neurosciences and Mental Health, The Hospital for Sick Children, Toronto, ON Canada; 29 Department of Medical Biophysics, University of Toronto, Toronto, ON Canada; 30 Department of Neuropsychiatry, Universidade Federal de Santa Maria, Santa Maria, Brazil; 31 Psychiatric Genetics, QIMR Berghofer Medical Research Institute, Brisbane, QLD Australia; 32 Behavioural Genetics Clinic, Adult Autism Service, Behavioural and Developmental Psychiatry Clinical Academic Group, South London and Maudsley Foundation NHS Trust, London, UK; 33 The Sackler Institute for Translational Neurodevelopment, Institute of Psychiatry, Psychology & Neuroscience, King’s College London, London, UK; 34 Institute of Psychiatry, Psychology and Neuroscience, Kings College London, London, UK; 35 Diagnostic Imaging Research, The Hospital for Sick Children, University of Toronto, Toronto, ON Canada; 36 Department of Speech, Language, and Hearing Sciences, The George Washington University, Washington, DC USA; 37 Donders Institute for Brain, Cognition and Behaviour, Radboud University, Nijmegen, The Netherlands

**Keywords:** Neuroscience, Autism spectrum disorders

Correction to: *Nature Communications* 10.1038/s41467-019-13005-8, published online 31 October 2019.

The original version of this Article contained an error in Fig. 1, in which the images shown in panel 1b were inadvertently duplicated from those in panel 1a. This error was introduced during the preparation of figures during the revision process. This has been corrected in both the PDF and HTML versions of the Article.

