# Supplementary material for: Altered structural brain asymmetry in autism spectrum disorder in a study of 54 datasets
Source: Nat Commun. 2019 Oct 31;10:4958. doi: 10.1038/s41467-019-13005-8 (PMC6823355; doi:10.1038/s41467-019-13005-8)
Supplement: Supplementary file 1 — Supplementary Information [file 41467_2019_13005_MOESM1_ESM.pdf]

Postema et al.

Supplementary Information

Altered structural brain asymmetry in autism spectrum disorder in a study of 54 datasets

List of Tables:

Supplementary Table S1. Full linear mixed model results for the cortical thickness AIs 2

Supplementary Table 2. Full linear mixed model results for the cortical surface area AIs. 3

Supplementary Table 3. Full linear mixed model results for the subcortical volume AIs................... 4

Supplementary Table 4. Results of sensitivity analyses for cortical thickness AIs. 5

Supplementary Table 5. Results of sensitivity analyses for cortical surface area AIs......................... 7

Supplementary Table 6. Results of sensitivity analyses in subcortical volume AIs.. 9

Supplementary Table 7. Sex:diagnosis interaction effects for cortical thickness AIs. 10

Supplementary Table 8. Sex:diagnosis interaction effects for cortical surface area AIs. 12

Supplementary Table 9. Sex:diagnosis interaction effects for subcortical volume AIs. 14

Supplementary Table 10. Age:diagnosis interaction effects for cortical thickness AIs**.** 15

Supplementary Table 11. Age:diagnosis interaction effects for cortical surface area AIs**.** 17

Supplementary Table 12. Age:diagnosis interaction effects for subcortical volume AIs.. 19

Supplementary Table 13. Association of IQ with asymmetry within cases and within controls........ 20

Supplementary Table 14. Association of $\log_{10}$-normalized ADOS severity scores with brain asymmetry within cases... 21

Supplementary Table 15 Association of medication use with brain asymmetry within cases 22

List of Figures:

Supplementary Figure 1. Distributions of ADOS severity scores within cases, and of sex, age and IQ in both cases and controls. 23

Supplementary Figure 2. Joyplot of the distributions of AIs in the total sample (without winsorization).. 24

Supplementary Figure 3 Residual plots of the linear mixed effects model analysis of cortical thickness AIs and the AI of the total average cortical thickness 25

Supplementary Figure 4. Residual plots of the linear mixed effects model analysis of cortical surface area AIs and the AI of the total cortical surface area. 26

Supplementary Figure 5. Residual plots of the linear mixed effects model analysis of subcortical volume AIs and the AI of the lateral ventricles.. 27

Supplementary Figure 6. Correlations between AIs of cortical thickness. 28

Supplementary Figure 7. Correlations between AIs of cortical surface areas. 29

Supplementary Figure 8. Correlations between AIs of subcortical volumes. 30

Supplementary Figure 9. Scatter plots of the relationships between age and AIs of the total and regional cortical thicknesses. 31

Supplementary Figure 10. Scatter plots of the relationships between age and AIs of the total and regional cortical surface areas. 32

Supplementary Figure 11. Scatter plots of the relationships between age and AIs of subcortical volumes and lateral ventricles. 33

Supplementary Table S1. Full linear mixed model results for the cortical thickness AIs

AI region	N cases/ controls	β-value			Standard Error			*t*-value			*P*-value[1]			Cohen's *d* (95% CI)
		diag	age	sex	diag	age	sex	diag	age	sex	diag	age	sex	
bankssts thickness	1691/1750	0.00009	0.00002	0.00298	0.0014	0.0001	0.0019	0.06	0.20	1.58	0.950	0.845	0.113	0.002(-0.06,0.07)
caudalanteriorcingulate thickness	1699/1763	-0.00004	-0.00006	0.00234	0.0018	0.0001	0.0024	-0.02	-0.44	0.98	0.982	0.657	0.328	-0.001(-0.07,0.07)
caudalmiddlefrontal thickness	1705/1770	-0.00166	0.00003	-0.00046	0.0010	0.0001	0.0013	-1.72	0.32	-0.36	0.086	0.749	0.717	-0.059(-0.13,0.01)
cuneus thickness	1700/1767	-0.00215	0.00006	-0.00180	0.0012	0.0001	0.0015	-1.84	0.66	-1.17	0.066	0.508	0.240	-0.063(-0.13,0)
entorhinal thickness	1688/1752	0.00360	-0.00032	-0.00190	0.0022	0.0001	0.0028	1.67	-2.17	-0.68	0.095	**0.030**	0.495	0.057(-0.01,0.12)
frontalpole thickness	1704/1765	-0.00069	-0.00022	-0.00085	0.0023	0.0002	0.0030	-0.30	-1.37	-0.28	0.765	0.171	0.777	-0.01(-0.08,0.06)
fusiform thickness	1704/1767	0.00277	-0.00001	0.00234	0.0009	0.0001	0.0011	3.20	-0.17	2.06	***0.001***	0.862	**0.040**	0.109(0.04,0.18)
inferiorparietal thickness	1705/1769	-0.00146	-0.00002	-0.00089	0.0008	0.0001	0.0010	-1.85	-0.28	-0.86	0.065	0.782	0.392	-0.063(-0.13,0)
inferiortemporal thickness	1703/1768	0.00305	0.00001	0.00098	0.0010	0.0001	0.0014	2.97	0.07	0.73	***0.003***	0.948	0.467	0.102(0.03,0.17)
insula thickness	1700/1764	0.00107	0.00011	0.00256	0.0009	0.0001	0.0012	1.15	1.41	2.10	0.252	0.158	**0.036**	0.039(-0.03,0.11)
isthmuscingulate thickness	1699/1769	-0.00365	0.00027	0.00186	0.0014	0.0001	0.0018	-2.58	2.81	1.02	***0.010***	**0.005**	0.310	-0.088(-0.16,-0.02)
lateraloccipital thickness	1701/1766	0.00075	0.00009	0.00119	0.0008	0.0001	0.0011	0.92	1.40	1.12	0.359	0.160	0.264	0.031(-0.04,0.1)
lateralorbitofrontal thickness	1704/1771	0.00017	-0.00003	-0.00066	0.0010	0.0001	0.0014	0.16	-0.33	-0.48	0.872	0.739	0.631	0.006(-0.06,0.07)
lingual thickness	1704/1769	0.00051	0.00000	-0.00379	0.0010	0.0001	0.0013	0.53	0.01	-3.03	0.594	0.988	**0.002**	0.018(-0.05,0.08)
medialorbitofrontal thickness	1705/1769	-0.00442	0.00043	-0.00052	0.0013	0.0001	0.0017	-3.47	4.23	-0.31	***0.001***	**0.00002**	0.758	-0.119(-0.19,-0.05)
middletemporal thickness	1702/1764	-0.00034	0.00004	0.00106	0.0010	0.0001	0.0013	-0.35	0.47	0.84	0.726	0.641	0.403	-0.012(-0.08,0.05)
paracentral thickness	1702/1771	0.00114	0.00002	-0.00037	0.0010	0.0001	0.0012	1.20	0.29	-0.29	0.231	0.772	0.769	0.041(-0.03,0.11)
parahippocampal thickness	1702/1764	-0.00028	0.00004	-0.00264	0.0016	0.0001	0.0020	-0.18	0.36	-1.31	0.856	0.717	0.190	-0.006(-0.07,0.06)
parsopercularis thickness	1703/1769	-0.00059	0.00001	0.00083	0.0010	0.0001	0.0014	-0.57	0.13	0.61	0.568	0.898	0.540	-0.02(-0.09,0.05)
parsorbitalis thickness	1705/1770	-0.00348	0.00005	0.00139	0.0015	0.0001	0.0020	-2.26	0.43	0.69	**0.024**	0.670	0.491	-0.077(-0.14,-0.01)
parstriangularis thickness	1703/1769	-0.00035	-0.00009	0.00144	0.0011	0.0001	0.0015	-0.31	-0.98	0.96	0.759	0.330	0.336	-0.011(-0.08,0.06)
pericalcarine thickness	1699/1768	-0.00086	-0.00013	0.00063	0.0014	0.0001	0.0018	-0.62	-1.12	0.34	0.536	0.262	0.731	-0.021(-0.09,0.05)
postcentral thickness	1704/1766	-0.00107	0.00007	-0.00040	0.0009	0.0001	0.0011	-1.25	1.16	-0.36	0.211	0.245	0.720	-0.043(-0.11,0.02)
posteriorcingulate thickness	1706/1765	-0.00251	0.00007	-0.00079	0.0012	0.0001	0.0015	-2.13	0.80	-0.51	**0.034**	0.423	0.611	-0.073(-0.14,-0.01)
precentral thickness	1704/1766	-0.00045	-0.00009	-0.00038	0.0007	0.0001	0.0010	-0.60	-1.52	-0.39	0.547	0.129	0.700	-0.021(-0.09,0.05)
precuneus thickness	1705/1769	-0.00027	0.00005	-0.00188	0.0007	0.0001	0.0010	-0.37	0.81	-1.96	0.710	0.418	0.050	-0.013(-0.08,0.05)
rostralanteriorcingulate thickness	1700/1765	-0.00568	-0.00010	0.00321	0.0017	0.0001	0.0022	-3.37	-0.69	1.45	***0.001***	0.491	0.147	-0.116(-0.18,-0.05)
rostralmiddlefrontal thickness	1707/1769	-0.00255	-0.00022	0.00169	0.0009	0.0001	0.0011	-2.94	-2.87	1.47	***0.003***	**0.004**	0.141	-0.101(-0.17,-0.03)
superiorfrontal thickness	1706/1771	-0.00251	0.00003	-0.00116	0.0006	0.0001	0.0008	-3.92	0.48	-1.38	***0.0001***	0.630	0.169	-0.134(-0.2,-0.07)
superiorparietal thickness	1702/1769	0.00023	0.00005	-0.00001	0.0007	0.0001	0.0009	0.35	0.89	-0.01	0.725	0.372	0.995	0.012(-0.05,0.08)
superiortemporal thickness	1693/1760	-0.00168	0.00003	0.00177	0.0009	0.0001	0.0011	-1.97	0.47	1.59	**0.049**	0.635	0.112	-0.068(-0.13,0)
supramarginal thickness	1698/1769	0.00002	-0.00009	0.00187	0.0009	0.0001	0.0011	0.03	-1.35	1.63	0.980	0.177	0.103	0.001(-0.07,0.07)
temporalpole thickness	1697/1761	0.00300	-0.00002	0.00052	0.0020	0.0002	0.0026	1.49	-0.12	0.20	0.136	0.906	0.844	0.051(-0.02,0.12)
transversetemporal thickness	1704/1771	0.00152	0.00025	0.00107	0.0017	0.0001	0.0022	0.91	1.95	0.49	0.364	0.051	0.626	0.031(-0.04,0.1)
Total average thickness	1706/1771	-0.00061	0.00001	0.00021	0.0003	0.0000	0.0004	-1.96	0.33	0.52	0.050	0.742	0.602	-0.067(-0.13,0)

[1]Unadjusted *P* values are shown, with in **bold** those that are significant ($P < 0.05$) at the uncorrected level. Those p-values for diagnosis that survived multiple testing correction (FDR < 0.05) are indicated in *italic.*

Supplementary Table 2. Full linear mixed model results for the cortical surface area AIs.

AI region	N cases/ controls	β-value			Standard Error			*t*-value			*P*-value[1]			Cohen's *d* (95% CI)
		diag	age	sex	diag	age	sex	diag	age	sex	diag	age	sex	
bankssts surface area	1685/1745	0.00292	-0.00081	-0.00133	0.0030	0.0002	0.0038	0.97	-4.69	-0.35	0.332	**0.000003**	0.729	0.033(-0.03,0.1)
caudalanteriorcingulate surface area	1701/1752	-0.00176	-0.00043	0.00742	0.0040	0.0002	0.0052	-0.44	-1.73	1.43	0.662	0.084	0.151	-0.015(-0.08,0.05)
caudalmiddlefrontal surface area	1704/1763	-0.00186	0.00010	0.00642	0.0027	0.0002	0.0034	-0.70	0.65	1.89	0.483	0.516	0.059	-0.024(-0.09,0.04)
cuneus surface area	1700/1754	-0.00303	0.00000	-0.00212	0.0024	0.0002	0.0032	-1.24	0.00	-0.66	0.215	0.998	0.506	-0.043(-0.11,0.02)
entorhinal surface area	1687/1733	-0.00687	0.00054	-0.00009	0.0043	0.0003	0.0055	-1.61	1.96	-0.02	0.108	0.050	0.986	-0.055(-0.12,0.01)
frontalpole surface area	1707/1757	0.00276	0.00014	0.00351	0.0032	0.0002	0.0042	0.86	0.58	0.84	0.388	0.560	0.400	0.03(-0.04,0.1)
fusiform surface area	1702/1760	-0.00477	0.00007	0.00416	0.0018	0.0001	0.0024	-2.58	0.56	1.74	**0.010**	0.574	0.082	-0.089(-0.16,-0.02)
inferiorparietal surface area	1705/1758	0.00034	-0.00009	0.00072	0.0019	0.0001	0.0024	0.18	-0.74	0.30	0.857	0.461	0.767	0.006(-0.06,0.07)
inferiortemporal surface area	1698/1757	-0.00114	0.00006	-0.00066	0.0021	0.0001	0.0027	-0.55	0.45	-0.24	0.585	0.654	0.807	-0.019(-0.09,0.05)
insula surface area	1699/1751	0.00063	-0.00004	-0.00279	0.0016	0.0001	0.0022	0.38	-0.34	-1.29	0.701	0.733	0.197	0.013(-0.05,0.08)
isthmuscingulate surface area	1699/1753	0.00134	0.00006	0.00048	0.0026	0.0002	0.0034	0.51	0.34	0.14	0.611	0.734	0.888	0.017(-0.05,0.08)
lateraloccipital surface area	1705/1754	-0.00080	-0.00009	0.00298	0.0018	0.0001	0.0023	-0.45	-0.80	1.30	0.654	0.424	0.193	-0.015(-0.08,0.05)
lateralorbitofrontal surface area	1701/1762	-0.00500	0.00010	-0.00013	0.0015	0.0001	0.0020	-3.28	0.81	-0.07	***0.001***	0.421	0.946	-0.112(-0.18,-0.05)
lingual surface area	1696/1758	0.00046	0.00017	0.00286	0.0020	0.0001	0.0026	0.23	1.14	1.08	0.820	0.252	0.279	0.008(-0.06,0.07)
medialorbitofrontal surface area	1704/1763	0.00675	-0.00005	-0.00243	0.0021	0.0002	0.0028	3.17	-0.27	-0.87	***0.002***	0.787	0.385	0.109(0.04,0.18)
middletemporal surface area	1698/1757	-0.00073	-0.00007	0.00166	0.0017	0.0001	0.0021	-0.44	-0.64	0.77	0.661	0.522	0.441	-0.015(-0.08,0.05)
paracentral surface area	1703/1762	0.00408	-0.00018	0.00104	0.0023	0.0001	0.0029	1.81	-1.29	0.36	0.071	0.195	0.720	0.062(0,0.13)
parahippocampal surface area	1692/1752	-0.00051	-0.00008	0.00168	0.0028	0.0002	0.0036	-0.19	-0.47	0.47	0.853	0.639	0.637	-0.006(-0.07,0.06)
parsopercularis surface area	1704/1752	0.00042	0.00016	0.00050	0.0028	0.0002	0.0036	0.15	1.00	0.14	0.880	0.317	0.890	0.005(-0.06,0.07)
parsorbitalis surface area	1699/1760	0.00321	0.00008	0.00054	0.0021	0.0001	0.0028	1.50	0.60	0.20	0.133	0.551	0.844	0.052(-0.02,0.12)
parstriangularis surface area	1702/1758	0.00024	-0.00018	0.00459	0.0027	0.0002	0.0035	0.09	-1.02	1.32	0.929	0.309	0.188	0.003(-0.06,0.07)
pericalcarine surface area	1699/1762	-0.00134	-0.00004	0.00129	0.0023	0.0002	0.0030	-0.58	-0.22	0.43	0.561	0.825	0.670	-0.02(-0.09,0.05)
postcentral surface area	1706/1759	0.00135	0.00002	0.00082	0.0017	0.0001	0.0022	0.79	0.18	0.37	0.430	0.855	0.709	0.027(-0.04,0.09)
posteriorcingulate surface area	1703/1758	-0.00189	-0.00023	0.00406	0.0026	0.0002	0.0034	-0.72	-1.41	1.20	0.475	0.160	0.232	-0.025(-0.09,0.04)
precentral surface area	1703/1757	-0.00203	-0.00012	-0.00102	0.0014	0.0001	0.0018	-1.43	-1.38	-0.56	0.154	0.168	0.578	-0.049(-0.12,0.02)
precuneus surface area	1702/1762	-0.00017	0.00009	0.00038	0.0016	0.0001	0.0020	-0.11	1.02	0.19	0.915	0.310	0.851	-0.004(-0.07,0.06)
rostralanteriorcingulate surface area	1701/1760	-0.00344	0.00009	0.00583	0.0034	0.0003	0.0045	-1.00	0.36	1.30	0.317	0.719	0.193	-0.034(-0.1,0.03)
rostralmiddlefrontal surface area	1705/1761	0.00061	0.00000	-0.00140	0.0016	0.0001	0.0020	0.39	0.01	-0.69	0.694	0.990	0.489	0.013(-0.05,0.08)
superiorfrontal surface area	1710/1763	0.00071	-0.00009	-0.00168	0.0012	0.0001	0.0016	0.57	-1.18	-1.05	0.569	0.240	0.294	0.019(-0.05,0.09)
superiorparietal surface area	1702/1763	-0.00065	0.00002	-0.00233	0.0016	0.0001	0.0021	-0.40	0.16	-1.12	0.688	0.873	0.263	-0.014(-0.08,0.05)
superiortemporal surface area	1691/1750	-0.00145	-0.00004	-0.00152	0.0015	0.0001	0.0020	-0.96	-0.34	-0.77	0.339	0.733	0.440	-0.033(-0.1,0.03)
supramarginal surface area	1698/1759	0.00013	-0.00003	-0.00519	0.0022	0.0002	0.0029	0.06	-0.21	-1.80	0.954	0.831	0.072	0.002(-0.06,0.07)
temporalpole surface area	1705/1751	0.00191	0.00050	0.00262	0.0030	0.0002	0.0039	0.65	2.29	0.68	0.517	**0.022**	0.497	0.022(-0.04,0.09)
transversetemporal surface area	1704/1757	0.00046	-0.00023	-0.00068	0.0028	0.0002	0.0036	0.16	-1.23	-0.19	0.870	0.219	0.850	0.006(-0.06,0.07)
Total surface area	1706/1763	-0.00037	-0.00002	0.00008	0.0004	0.0000	0.0005	-0.95	-0.71	0.16	0.344	0.480	0.876	-0.032(-0.1,0.03)

[1]Unadjusted *P* values are shown, with in **bold** those that are significant ($P < 0.05$) at the uncorrected level. Those p-values for diagnosis that survived multiple testing correction (FDR < 0.05) are indicated in *italic.*

Supplementary Table 3. Full linear mixed model results for the subcortical volume AIs.

	N cases/	β-value			Standard Error			*t*-value			*P*-value[1]			
**AI region**	**controls**	**diag**	**age**	**sex**	**diag**	**age**	**sex**	**diag**	**age**	**sex**	**diag**	**age**	**sex**	**Cohen's *d* (95% CI)**
Accumbens	1716/1762	-0.00220	-0.00026	-0.00022	0.0028	0.0002	0.0036	-0.80	-1.09	-0.06	0.425	0.276	0.952	-0.027(-0.09,0.04)
Amygdala	1709/1768	0.00018	0.00002	0.00513	0.0022	0.0002	0.0029	0.08	0.13	1.77	0.934	0.893	0.077	0.003(-0.06,0.07)
CaudateNucleus	1711/1763	0.00293	0.00031	0.00223	0.0013	0.0001	0.0017	2.24	2.70	1.29	**0.025**	**0.007**	0.196	0.077(0.01,0.14)
GlobusPallidus	1707/1761	0.00113	-0.00053	0.00107	0.0021	0.0002	0.0028	0.53	-2.88	0.38	0.593	**0.004**	0.702	0.018(-0.05,0.08)
Hippocampus	1708/1762	-0.00106	0.00002	-0.00017	0.0015	0.0001	0.0020	-0.70	0.14	-0.08	0.485	0.885	0.934	-0.024(-0.09,0.04)
LateralVentricle	1660/1727	-0.00169	-0.00016	0.00306	0.0051	0.0003	0.0065	-0.33	-0.52	0.47	0.741	0.601	0.637	-0.011(-0.08,0.06)
Putamen	1712/1763	0.00395	0.00049	0.00273	0.0012	0.0001	0.0015	3.40	5.03	1.77	***0.001***	**$5.2\cdot10^{-7}$**	0.076	0.116(0.05,0.18)
Thalamus	1690/1763	0.00126	0.00063	0.00255	0.0011	0.0001	0.0015	1.15	6.36	1.75	0.252	**$2.3\cdot10^{-10}$**	0.081	0.039(-0.03,0.11)

[1]Unadjusted *P* values are shown, with in **bold** those that are significant ($P < 0.05$) at the uncorrected level. Those p-values for diagnosis that survived multiple testing correction (FDR < 0.05) are indicated in *italic.*

Supplementary Table 4. Results of sensitivity analyses for cortical thickness AIs. Results are shown (1) after outliers were winsorized, (2) after a non-linear effect of age was added to the model, (3) after examining the subset of 3T acquired data, (4) after removing subjects below 6 year of age, and (5) after removing subjects aged 40 years or older.

	**winsorised**		**non-linear age**		**3T**		**age ≥ 6y**		**age < 40y**	
**AI region**	**P[1]**	***d***	**P[1]**	***d***	**P[1]**	***d***	**P[1]**	***d***	**P[1]**	***d***
bankssts thickness	0.956	0.002	0.978	0.00	0.762	0.01	0.849	0.01	0.952	0.00
caudalanteriorcingulate thickness	0.986	-0.001	0.965	0.00	0.578	0.02	0.769	0.01	0.867	-0.01
caudalmiddlefrontal thickness	0.077	-0.06	0.090	-0.06	0.210	-0.05	0.149	-0.05	0.149	-0.05
cuneus thickness	0.067	-0.06	0.065	-0.06	0.068	-0.07	0.093	-0.06	0.066	-0.06
entorhinal thickness	0.091	0.06	0.103	0.06	0.287	0.04	0.184	0.05	0.096	0.06
frontalpole thickness	0.761	-0.01	0.747	-0.01	0.979	0.00	0.914	0.00	0.786	-0.01
fusiform thickness	***0.002***	0.11	***0.002***	0.11	***0.006***	0.10	***0.004***	0.10	***0.002***	0.11
inferiorparietal thickness	0.052	-0.07	0.059	-0.06	0.131	-0.06	0.091	-0.06	**0.039**	-0.07
inferiortemporal thickness	***0.003***	0.10	***0.003***	0.10	**0.016**	0.09	***0.004***	0.10	***0.002***	0.11
insula thickness	0.259	0.04	0.262	0.04	0.303	0.04	0.231	0.04	0.224	0.04
isthmuscingulate thickness	***0.010***	-0.09	***0.010***	-0.09	0.096	-0.06	**0.015**	-0.09	***0.006***	-0.10
lateraloccipital thickness	0.355	0.03	0.368	0.03	0.459	0.03	0.308	0.04	0.462	0.03
lateralorbitofrontal thickness	0.980	0.00	0.855	0.01	0.874	-0.01	0.944	0.00	0.996	0.00
lingual thickness	0.625	0.02	0.607	0.02	0.316	0.04	0.759	0.01	0.633	0.02
medialorbitofrontal thickness	***0.001***	-0.12	***0.001***	-0.12	***$4.5\cdot10^{-4}$***	-0.13	***$3.1\cdot10^{-4}$***	-0.13	***$3.2\cdot10^{-4}$***	-0.13
middletemporal thickness	0.684	-0.01	0.721	-0.01	0.530	-0.02	0.410	-0.03	0.829	-0.01
paracentral thickness	0.204	0.04	0.231	0.04	0.116	0.06	0.247	0.04	0.325	0.03
parahippocampal thickness	0.866	-0.01	0.835	-0.01	0.740	-0.01	0.940	0.00	0.930	0.00
parsopercularis thickness	0.582	-0.02	0.584	-0.02	0.583	-0.02	0.601	-0.02	0.566	-0.02
parsorbitalis thickness	**0.024**	-0.08	**0.024**	-0.08	**0.015**	-0.09	**0.021**	-0.08	**0.021**	-0.08
parstriangularis thickness	0.746	-0.01	0.777	-0.01	0.746	-0.01	0.705	-0.01	0.682	-0.01
pericalcarine thickness	0.544	-0.02	0.540	-0.02	0.854	0.01	0.829	-0.01	0.508	-0.02
postcentral thickness	0.205	-0.04	0.197	-0.04	0.384	-0.03	0.309	-0.04	0.193	-0.05
posteriorcingulate thickness	0.043	-0.07	**0.036**	-0.07	0.123	-0.06	**0.016**	-0.09	**0.023**	-0.08
precentral thickness	0.482	-0.02	0.535	-0.02	0.763	-0.01	0.701	-0.01	0.774	-0.01
precuneus thickness	0.744	-0.01	0.711	-0.01	0.931	0.00	0.858	-0.01	0.638	-0.02
rostralanteriorcingulate thickness	***0.001***	-0.12	***0.001***	-0.12	***$8.7\cdot10^{-5}$***	-0.15	***$4.3\cdot10^{-4}$***	-0.12	***$3.3\cdot10^{-4}$***	-0.13

	winsorised		non-linear age		3T		age ≥ 6y		age < 40y	
**AI region**	**P[1]**	***d***	**P[1]**	***d***	**P[1]**	***d***	**P[1]**	***d***	**P[1]**	***d***
rostralmiddlefrontal thickness	***0.003***	-0.10	***0.004***	-0.10	***0.001***	-0.12	***0.001***	-0.12	***0.005***	-0.10
superiorfrontal thickness	***$8.7 \cdot 10^{-5}$***	-0.13	***$1 \cdot 10^{-4}$***	-0.13	***$7.4 \cdot 10^{-6}$***	-0.17	***$5.9 \cdot 10^{-5}$***	-0.14	***$7.2 \cdot 10^{-5}$***	-0.14
superiorparietal thickness	0.736	0.01	0.740	0.01	0.498	0.03	0.663	0.02	0.586	0.02
superiortemporal thickness	**0.049**	-0.07	**0.049**	-0.07	***0.004***	-0.11	0.051	-0.07	0.058	-0.07
supramarginal thickness	0.971	0.001	0.972	0.00	0.912	0.00	0.816	0.01	0.976	0.00
temporalpole thickness	0.137	0.05	0.137	0.05	0.226	0.05	0.117	0.06	0.082	0.06
transversetemporal thickness	0.374	0.03	0.375	0.03	0.504	0.03	0.701	0.01	0.303	0.04
Total average thickness	0.051	-0.07	**0.050**	-0.07	**0.039**	-0.08	**0.045**	-0.07	0.052	-0.07

[1]Unadjusted *P* values are shown, with in **bold** those that are significant (*P* < 0.05) at the uncorrected level. Those p-values for diagnosis that survived multiple testing correction (FDR < 0.05) are indicated in *italic.*

Supplementary Table 5. Results of sensitivity analyses for cortical surface area AIs. Results are shown (1) after outliers were winsorized, (2) after a non-linear effect of age was added to the model, (3) after examining the subset of 3T acquired data, (4) after removing subjects below 6 year of age, and (5) after removing subjects aged 40 year or older.

	**winsorised**		**non-linear age**		**3T**		**age ≥ 6y**		**age < 40y**	
**AI region**	**P[1]**	***d***	**P[1]**	***d***	**P[1]**	***d***	**P[1]**	***d***	**P[1]**	***d***
bankssts surface area	0.311	0.04	0.356	0.03	0.590	0.02	0.454	0.03	0.357	0.03
caudalanteriorcingulate surface area	0.543	-0.02	0.643	-0.02	0.818	0.01	0.886	-0.01	0.636	-0.02
caudalmiddlefrontal surface area	0.418	-0.03	0.500	-0.02	0.328	-0.04	0.232	-0.04	0.471	-0.03
cuneus surface area	0.222	-0.04	0.232	-0.04	0.130	-0.06	0.309	-0.04	0.128	-0.05
entorhinal surface area	0.117	-0.05	0.108	-0.06	**0.029**	-0.08	0.118	-0.06	0.153	-0.05
frontalpole surface area	0.410	0.03	0.408	0.03	0.107	0.06	0.692	0.01	0.366	0.03
fusiform surface area	**0.011**	-0.09	**0.010**	-0.09	***0.001***	-0.13	***0.002***	-0.11	**0.007**	-0.09
inferiorparietal surface area	0.857	0.01	0.874	0.01	0.905	0.00	0.811	-0.01	0.961	0.00
inferiortemporal surface area	0.594	-0.02	0.591	-0.02	0.519	-0.02	0.773	-0.01	0.420	-0.03
insula surface area	0.675	0.01	0.703	0.01	0.508	0.03	0.592	0.02	0.741	0.01
isthmuscingulate surface area	0.514	0.02	0.661	0.02	0.549	0.02	0.752	0.01	0.534	0.02
lateraloccipital surface area	0.667	-0.02	0.620	-0.02	0.505	0.03	0.841	-0.01	0.776	-0.01
lateralorbitofrontal surface area	***0.001***	-0.11	***0.001***	-0.11	***0.001***	-0.12	***0.001***	-0.12	***0.001***	-0.12
lingual surface area	0.762	0.01	0.822	0.01	0.744	0.01	0.934	0.00	0.721	0.01
medialorbitofrontal surface area	**0.003**	0.10	***0.002***	0.11	***0.001***	0.12	***0.002***	0.11	***0.001***	0.12
middletemporal surface area	0.682	-0.01	0.672	-0.01	0.647	-0.02	0.850	-0.01	0.628	-0.02
paracentral surface area	0.070	0.06	0.065	0.06	**0.047**	0.07	**0.048**	0.07	0.102	0.06
parahippocampal surface area	0.808	-0.01	0.839	-0.01	0.960	0.00	0.947	0.00	0.620	-0.02
parsopercularis surface area	0.895	0.01	0.932	0.00	0.945	0.00	0.740	0.01	0.680	0.01
parsorbitalis surface area	0.143	0.05	0.144	0.05	0.207	0.05	0.099	0.06	0.196	0.05
parstriangularis surface area	0.932	0.00	0.963	0.00	0.549	-0.02	0.992	0.00	0.668	0.02
pericalcarine surface area	0.584	-0.02	0.562	-0.02	0.220	-0.05	0.476	-0.03	0.557	-0.02
postcentral surface area	0.437	0.03	0.439	0.03	0.763	0.01	0.415	0.03	0.486	0.02
posteriorcingulate surface area	0.466	-0.03	0.448	-0.03	0.808	-0.01	0.683	-0.01	0.486	-0.02

	**winsorised**		**non-linear age**		**3T**		**age ≥ 6y**		**age < 40y**	
**AI region**	**P[1]**	***d***	**P[1]**	***d***	**P[1]**	***d***	**P[1]**	***d***	**P[1]**	***d***
precentral surface area	0.150	-0.05	0.149	-0.05	**0.030**	-0.08	0.059	-0.07	0.145	-0.05
precuneus surface area	0.950	0.00	0.935	0.00	0.674	-0.02	0.601	-0.02	0.978	0.00
rostralanteriorcingulate surface area	0.329	-0.03	0.314	-0.03	0.323	-0.04	0.242	-0.04	0.245	-0.04
rostralmiddlefrontal surface area	0.641	0.02	0.697	0.01	0.550	0.02	0.805	0.01	0.644	0.02
superiorfrontal surface area	0.670	0.02	0.511	0.02	0.986	0.00	0.681	0.01	0.411	0.03
superiorparietal surface area	0.717	-0.01	0.635	-0.02	0.779	-0.01	0.726	-0.01	0.609	-0.02
superiortemporal surface area	0.356	-0.03	0.314	-0.03	0.215	-0.05	0.320	-0.04	0.603	-0.02
supramarginal surface area	0.963	0.00	0.992	0.00	0.475	0.03	0.849	0.01	0.803	0.01
temporalpole surface area	0.555	0.02	0.510	0.02	0.840	0.01	0.639	0.02	0.497	0.02
transversetemporal surface area	0.887	0.01	0.864	0.01	0.937	0.00	0.901	0.00	0.906	0.00
totalsurf	0.431	-0.03	0.321	-0.03	0.196	-0.05	0.244	-0.04	0.411	-0.03

.[1]Unadjusted *P* values are shown,, with in **bold** those that are significant (*P* < 0.05) at the uncorrected level. Those p-values for diagnosis that survived multiple testing correction (FDR < 0.05) are indicated in *italic.*

Supplementary Table 6. Results of sensitivity analyses in subcortical volume AIs. Results are shown (1) after outliers were winsorized, (2) after a non-linear effect of age was added to the model, (3) after examining the subset of 3T acquired data, (4) after removing subjects below 6 year of age, and (5) after removing subjects aged 40 year or older.

	winsorised		non-linear age		3T		age ≥ 6y		age < 40y	
**AI region**	**P[1]**	***d***	**P[1]**	***d***	**P[1]**	***d***	**P[1]**	***d***	**P[1]**	***d***
Accumbens	0.386	-0.030	0.445	-0.026	0.560	-0.022	0.271	-0.039	0.544	-0.021
Amygdala	0.896	0.004	0.921	0.003	0.753	0.012	0.935	-0.003	0.765	-0.010
CaudateNucleus	**0.035**	0.072	**0.027**	0.076	***0.008***	0.099	0.014	0.086	0.031	0.075
GlobusPallidus	0.666	0.015	0.640	0.016	0.300	0.039	0.446	0.027	0.844	0.007
Hippocampus	0.527	-0.022	0.504	-0.023	0.233	-0.044	0.455	-0.026	0.535	-0.022
LateralVentricle	0.741	-0.011	0.711	-0.013	0.988	-0.001	0.672	-0.015	0.759	-0.011
Putamen*	***0.001***	0.118	***0.001***	0.117	***0.001***	0.125	***0.001***	0.121	***0.002***	0.106
Thalamus	0.251	0.039	0.217	0.042	**0.037**	0.078	0.134	0.053	0.104	0.057

.[1]Unadjusted *P* values are shown,, with in **bold** those that are significant (*P* < 0.05) at the uncorrected level. Those p-values for diagnosis that survived multiple testing correction (FDR < 0.05) are also indicated in *italic.*

Supplementary Table 7. Sex:diagnosis interaction effects for cortical thickness AIs. P-values and Cohen's *d* effect sizes for the main effects of diagnosis are shown for the primary and sex:diagnosis interaction models, as well as for the stratification analyses separately in males and females. P-values for the sex:diagnosis interaction effects are also shown. Additionally, for the primary and interaction models the AIC and BIC model fit measures are shown.

	**Primary analysis model**				**Sex:diagnosis interaction model**					**Males only**		**Females only**	
**region**	**P_{diag}[1]**	***d***	**AIC**	**BIC**	**P_{diag}[1]**	***d***	**$P_{diag*sex}$[1]**	**AIC**	**BIC**	**P_{diag}[1]**	***d***	**P_{diag}[1]**	***d***
bankssts thickness	0.950	0.002	-12107	-12070	0.672	-0.03	0.385	-12106	-12063	0.650	0.02	0.512	-0.05
caudalanteriorcingulate thickness	0.982	-0.001	-10511	-10474	0.859	-0.02	0.639	-10509	-10466	0.970	0.00	0.722	-0.03
caudalmiddlefrontal thickness	0.086	-0.06	-14975	-14938	0.447	-0.06	0.058	-14977	-14934	0.457	-0.03	**0.014**	-0.20
cuneus thickness	0.066	-0.06	-13627	-13590	0.339	-0.06	0.092	-13628	-13585	0.382	-0.03	**0.015**	-0.20
entorhinal thickness	0.095	0.06	-9348	-9311	0.087	-0.02	0.636	-9347	-9304	0.086	0.07	0.823	0.02
frontalpole thickness	0.765	-0.01	-8929	-8892	0.616	0.02	0.586	-8927	-8884	0.630	-0.02	0.499	0.06
fusiform thickness	***0.001***	0.11	-15724	-15687	***0.002***	-0.02	0.536	-15722	-15679	0.003	0.11	0.252	0.09
inferiorparietal thickness	0.065	-0.06	-16323	-16286	0.271	-0.05	0.172	-16322	-16279	0.289	-0.04	0.061	-0.15
inferiortemporal thickness	***0.003***	0.10	-14532	-14495	***0.002***	-0.03	0.314	-14531	-14488	***0.002***	0.12	0.663	0.04
insula thickness	0.252	0.04	-15213	-15176	**0.030**	-0.09	**0.007**	-15218	-15175	0.037	0.08	0.181	-0.11
isthmuscingulate thickness	***0.010***	-0.09	-12320	-12283	***0.006***	0.03	0.330	-12319	-12276	***0.007***	-0.10	0.666	-0.04
lateraloccipital thickness	0.359	0.03	-16137	-16100	0.240	-0.03	0.414	-16135	-16092	0.259	0.04	0.797	-0.02
lateralorbitofrontal thickness	0.872	0.01	-14413	-14376	0.930	0.02	0.576	-14411	-14368	0.930	0.00	0.783	0.02
lingual thickness	0.594	0.02	-15045	-15008	0.305	-0.04	0.198	-15044	-15001	0.353	0.04	0.342	-0.08
medialorbitofrontal thickness	***0.001***	-0.12	-13057	-13020	***0.0005***	0.03	0.420	-13055	-13012	***0.001***	-0.13	0.534	-0.05
middletemporal thickness	0.726	-0.01	-14979	-14942	0.567	0.02	0.546	-14978	-14934	0.535	-0.02	0.564	0.05
paracentral thickness	0.231	0.04	-15060	-15024	0.074	-0.06	0.095	-15061	-15018	0.080	0.07	0.171	-0.11
parahippocampal thickness	0.856	-0.01	-11609	-11572	0.558	0.03	0.322	-11608	-11565	0.533	-0.02	0.210	0.10
parsopercularis thickness	0.568	-0.02	-14483	-14446	0.888	-0.03	0.371	-14482	-14439	0.901	0.00	0.219	-0.10
parsorbitalis thickness	**0.024**	-0.08	-11735	-11698	0.112	-0.04	0.278	-11734	-11691	0.099	-0.06	0.100	-0.13
parstriangularis thickness	0.759	-0.01	-13813	-13776	0.468	0.04	0.288	-13812	-13769	0.426	-0.03	0.317	0.08
pericalcarine thickness	0.536	-0.02	-12416	-12379	0.807	-0.03	0.451	-12414	-12371	0.822	-0.01	0.287	-0.09
postcentral thickness	0.211	-0.04	-15802	-15765	0.462	-0.03	0.347	-15801	-15758	0.516	-0.02	0.194	-0.11
posteriorcingulate thickness	**0.034**	-0.07	-13588	-13551	**0.019**	0.03	0.326	-13587	-13543	0.023	-0.09	0.813	-0.02
precentral thickness	0.547	-0.02	-16743	-16706	0.779	-0.02	0.528	-16742	-16699	0.770	-0.01	0.655	-0.04

precuneus thickness	0.710	-0.01	-16940	-16903	0.599	-0.07	0.040	-16942	-16899	0.669	0.02	0.033	-0.17
rostralanteriorcingulate thickness	***0.001***	-0.12	-11088	-11051	***9.8·10^-6***	0.11	***0.001***	-11097	-11054	***1.4·10^-5***	-0.17	0.165	0.11
rostralmiddlefrontal thickness	***0.003***	-0.10	-15671	-15634	***0.003***	0.02	0.512	-15669	-15626	***0.003***	-0.11	0.382	-0.07
superiorfrontal thickness	***8.9·10^-5***	-0.13	-17795	-17758	***2.9·10^-5***	0.05	0.135	-17795	-17752	***1.5·10^-5***	-0.17	0.726	-0.03
superiorparietal thickness	0.725	0.01	-17583	-17546	0.197	-0.08	**0.021**	-17586	-17543	0.236	0.05	**0.045**	-0.16
superiortemporal thickness	**0.049**	-0.07	-15790	-15753	0.295	-0.06	0.079	-15791	-15748	0.292	-0.04	**0.017**	-0.20
supramarginal thickness	0.980	0.00	-15627	-15590	0.587	-0.04	0.217	-15627	-15583	0.516	0.02	0.245	-0.10
temporalpole thickness	0.136	0.05	-9867	-9830	0.116	-0.02	0.602	-9865	-9822	0.155	0.05	0.708	0.03
transversetemporal thickness	0.364	0.03	-11161	-11124	0.275	-0.02	0.526	-11159	-11116	0.336	0.04	0.973	0.003
Total average thickness	0.050	-0.07	-22896	-22859	0.249	-0.05	0.138	-22896	-22853	0.235	-0.05	**0.034**	-0.17

[1]Unadjusted *P* values are shown,, with in **bold** those that are significant (*P* < 0.05) at the uncorrected level. Those that survived multiple testing correction (FDR < 0.05) are also indicated in *italic.*

Supplementary Table 8. Sex:diagnosis interaction effects for cortical surface area AIs. P-values and Cohen's d effect sizes for the main effects of diagnosis are shown for the primary and sex:diagnosis interaction models, as well as for the stratification analyses separately in males and females. P-values for the sex:diagnosis interaction effects are also shown. Additionally, for the primary and interaction models the AIC and BIC model fit measures are shown. .

	**Primary analysis model**				**Sex:diagnosis interaction model**					**Males only**		**Females only**	
**region**	**P_{diag}[1]**	***d***	**AIC**	**BIC**	**P_{diag}[1]**	***d***	**$P_{diag*sex}$[1]**	**AIC**	**BIC**	**P_{diag}[1]**	***d***	**P_{diag}[1]**	***d***
bankssts surface area	0.332	0.03	-6952	-6915	0.565	0.02	0.488	-6951	-6908	0.586	0.02	0.331	0.08
caudalanteriorcingulate surface area	0.662	-0.02	-5027	-4990	0.858	-0.02	0.610	-5025	-4982	0.891	-0.01	0.480	-0.06
caudalmiddlefrontal surface area	0.483	-0.02	-7903	-7866	0.365	0.02	0.524	-7901	-7858	0.383	-0.03	0.818	0.02
cuneus surface area	0.215	-0.04	-8508	-8471	0.680	-0.06	0.087	-8509	-8466	0.721	-0.01	**0.019**	-0.20
entorhinal surface area	0.108	-0.06	-4612	-4576	0.180	-0.01	0.786	-4610	-4567	0.177	-0.05	0.242	-0.10
frontalpole surface area	0.388	0.03	-6670	-6633	0.412	-0.003	0.932	-6668	-6625	0.388	0.03	0.827	0.02
fusiform surface area	**0.010**	-0.09	-10454	-10417	**0.009**	0.02	0.534	-10452	-10409	**0.008**	-0.10	0.611	-0.04
inferiorparietal surface area	0.857	0.01	-10288	-10251	0.982	0.02	0.660	-10286	-10243	0.979	-0.001	0.527	0.05
inferiortemporal surface area	0.585	-0.02	-9610	-9573	0.947	-0.04	0.307	-9609	-9566	0.913	-0.004	0.214	-0.10
insula surface area	0.701	0.01	-11222	-11185	0.930	0.02	0.532	-11220	-11177	0.997	-0.0001	0.561	0.05
isthmuscingulate surface area	0.611	0.02	-7974	-7937	0.839	0.02	0.540	-7973	-7930	0.796	0.01	0.405	0.07
lateraloccipital surface area	0.654	-0.02	-10709	-10672	0.492	0.02	0.504	-10707	-10664	0.422	-0.03	0.729	0.03
lateralorbitofrontal surface area	***0.001***	-0.11	-11771	-11734	***0.001***	0.02	0.567	-11769	-11726	**0.003**	-0.12	0.166	-0.11
lingual surface area	0.820	0.01	-9826	-9789	0.949	0.02	0.517	-9825	-9782	0.904	-0.005	0.594	0.04
medialorbitofrontal surface area	***0.002***	0.11	-9475	-9438	**0.009**	0.02	0.526	-9474	-9431	**0.011**	0.10	**0.020**	0.19
middletemporal surface area	0.661	-0.02	-11137	-11100	0.891	-0.02	0.537	-11135	-11092	0.835	-0.01	0.559	-0.05
paracentral surface area	0.071	0.06	-9051	-9014	0.232	0.04	0.298	-9050	-9007	0.187	0.05	0.075	0.15
parahippocampal surface area	0.853	-0.01	-7631	-7594	0.990	-0.01	0.668	-7629	-7586	0.934	0.003	0.628	-0.04
parsopercularis surface area	0.880	0.01	-7494	-7457	0.912	0.02	0.564	-7492	-7449	0.982	0.001	0.628	0.04
parsorbitalis surface area	0.133	0.05	-9436	-9399	0.233	0.01	0.688	-9434	-9391	0.202	0.05	0.363	0.08
parstriangularis surface area	0.929	0.00	-7851	-7814	0.608	0.05	0.159	-7851	-7808	0.581	-0.02	0.182	0.11
pericalcarine surface area	0.561	-0.02	-8905	-8868	0.933	-0.05	0.143	-8905	-8862	0.940	0.00	0.070	-0.15
postcentral surface area	0.430	0.03	-10895	-10858	0.706	0.03	0.436	-10893	-10850	0.681	0.02	0.305	0.08
posteriorcingulate surface area	0.475	-0.02	-7924	-7887	0.252	0.04	0.241	-7923	-7880	0.265	-0.04	0.425	0.07
precentral surface area	0.154	-0.05	-12203	-12166	0.188	0.00	0.952	-12201	-12158	0.279	-0.04	0.622	-0.04

precuneus surface area	0.915	-0.004	-11488	-11451	0.760	0.02	0.626	-11486	-11443	0.708	-0.01	0.723	0.03
rostralanteriorcingulate surface area	0.317	-0.03	-6163	-6126	0.191	0.03	0.342	-6162	-6119	0.220	-0.05	0.633	0.04
rostralmiddlefrontal surface area	0.694	0.01	-11642	-11605	0.859	0.04	0.204	-11642	-11599	0.753	-0.01	0.187	0.11
superiorfrontal surface area	0.569	0.02	-13194	-13157	0.521	-0.01	0.766	-13192	-13149	0.508	0.03	0.951	0.01
superiorparietal surface area	0.688	-0.01	-11377	-11340	0.699	0.00	0.955	-11375	-11332	0.736	-0.01	0.901	-0.01
superiortemporal surface area	0.339	-0.03	-11787	-11750	0.244	0.02	0.478	-11786	-11743	0.259	-0.04	0.847	0.02
supramarginal surface area	0.954	0.002	-9191	-9154	0.806	0.02	0.478	-9190	-9147	0.838	-0.01	0.498	0.06
temporalpole surface area	0.517	0.02	-7204	-7168	0.350	-0.03	0.409	-7203	-7160	0.400	0.03	0.495	-0.06
transversetemporal surface area	0.870	0.01	-7600	-7563	0.908	0.003	0.939	-7598	-7555	0.958	0.00	0.937	0.01
totalsurf	0.344	-0.03	-21189	-21152	0.116	0.06	0.088	-21190	-21147	0.127	-0.06	0.269	0.09

[1]Unadjusted *P* values are shown, with in **bold** those that are significant (*P* < 0.05) at the uncorrected level. Those that survived multiple testing correction (FDR < 0.05) are also indicated in *italic.*

Supplementary Table 9. Sex:diagnosis interaction effects for subcortical volume AIs. P-values and Cohen's d effect sizes for the main effects of diagnosis are shown for the primary and sex:diagnosis interaction models, as well as for the stratification analyses separately in males and females. P-values for the sex:diagnosis interaction effects are also shown. Additionally, for the primary and interaction models the AIC and BIC model fit measures are shown.

	Primary analysis model				Sex:diagnosis interaction model					Males only		Females only	
**region**	P_{diag}[1]	*d*	AIC	BIC	P_{diag}[1]	*d*	$P_{diag*sex}$[1]	AIC	BIC	P_{diag}[1]	*d*	P_{diag}[1]	*d*
Accumbens	0.425	-0.03	-7684	-7647	0.231	0.04	0.26	-7683	-7640	0.234	-0.05	0.469	0.06
Amygdala	0.934	0.003	-9229	-9192	0.547	-0.04	0.21	-9229	-9186	0.561	0.02	0.167	-0.11
CaudateNucleus	**0.025**	0.08	-12825	-12788	***0.010***	-0.04	0.19	-12825	-12782	***0.011***	0.10	0.959	-0.004
GlobusPallidus	0.593	0.02	-9466	-9429	0.201	-0.06	0.06	-9467	-9424	0.204	0.05	0.209	-0.10
Hippocampus	0.485	-0.02	-11814	-11777	0.600	-0.01	0.79	-11812	-11769	0.579	-0.02	0.412	-0.07
LateralVentricle	0.741	-0.01	-3330	-3293	0.882	-0.04	0.30	-3329	-3286	0.879	0.01	0.264	-0.09
Putamen	***0.001***	0.12	-13668	-13632	***0.001***	-0.01	0.70	-13667	-13624	***0.001***	0.13	0.312	0.08
Thalamus	0.252	0.04	-13885	-13848	0.486	0.03	0.41	-13884	-13841	0.638	0.02	0.060	0.15

[1]Unadjusted *P* values are shown, with in **bold** those that are significant ($P < 0.05$) at the uncorrected level. Those that survived multiple testing correction (FDR < 0.05) are also indicated in *italic*

Supplementary Table 10. Age:diagnosis interaction effects for cortical thickness AIs. P-values and Cohen's *d* effect sizes for the main effects of diagnosis are shown for the primary and age:diagnosis interaction models, as well as for the stratification analyses separately in children and adults. P-values for the age:diagnosis interaction effects are also shown. Additionally, for both primary and interaction models the AIC and BIC model fit measures are shown.

	**Primary analysis model**				**Age:diagnosis interaction model**					**Children only**		**Adults only**	
**region**	**P_{diag}[1]**	***d***	**AIC**	**BIC**	**P_{diag}[1]**	***d***	**$P_{diag*age}$[1]**	**AIC**	**BIC**	**P_{diag}[1]**	***d***	**P_{diag}[1]**	***d***
bankssts thickness	0.950	0.002	-12107	-12070	0.819	0.01	0.767	-12105	-12062	0.542	-0.025	0.306	0.06
caudalanteriorcingulate thickness	0.982	-0.001	-10511	-10474	0.291	0.04	0.232	-10510	-10467	0.610	-0.021	0.402	0.05
caudalmiddlefrontal thickness	0.086	-0.06	-14975	-14938	0.840	-0.02	0.468	-14974	-14931	0.368	-0.037	0.078	-0.11
cuneus thickness	0.066	-0.06	-13627	-13590	0.179	0.02	0.607	-13625	-13582	0.087	-0.071	0.337	-0.06
entorhinal thickness	0.095	0.06	-9348	-9311	0.102	-0.03	0.348	-9347	-9304	0.288	0.044	0.131	0.10
frontalpole thickness	0.765	-0.01	-8929	-8892	0.864	-0.01	0.716	-8927	-8884	0.665	0.018	0.235	-0.07
fusiform thickness	***0.001***	0.11	-15724	-15687	**0.006**	-0.05	0.180	-15724	-15681	***0.001***	0.135	0.279	0.07
inferiorparietal thickness	0.065	-0.06	-16323	-16286	0.171	0.02	0.589	-16321	-16278	0.140	-0.061	0.313	-0.06
inferiortemporal thickness	***0.003***	0.10	-14532	-14495	**0.031**	-0.03	0.412	-14530	-14487	0.017	0.098	0.067	0.12
insula thickness	0.252	0.04	-15213	-15176	0.206	-0.03	0.418	-15211	-15168	0.212	0.051	0.833	0.01
isthmuscingulate thickness	***0.010***	-0.09	-12320	-12283	0.072	0.02	0.535	-12318	-12275	**0.011**	-0.104	0.342	-0.06
lateraloccipital thickness	0.359	0.03	-16137	-16100	0.650	-0.0003	0.992	-16135	-16092	0.319	0.041	0.832	0.01
lateralorbitofrontal thickness	0.872	0.01	-14413	-14376	0.558	0.03	0.447	-14411	-14368	0.998	0.000	0.808	0.02
lingual thickness	0.594	0.02	-15045	-15008	0.294	-0.03	0.366	-15044	-15000	0.351	0.038	0.804	-0.02
medialorbitofrontal thickness	***0.001***	-0.12	-13057	-13020	**0.023**	0.02	0.499	-13055	-13012	***0.001***	-0.143	0.241	-0.07
middletemporal thickness	0.726	-0.01	-14979	-14942	0.219	-0.05	0.109	-14980	-14937	0.576	0.023	0.112	-0.10
paracentral thickness	0.231	0.04	-15060	-15024	0.629	0.004	0.909	-15058	-15015	0.124	0.063	0.935	0.01
parahippocampal thickness	0.856	-0.01	-11609	-11572	0.258	-0.05	0.162	-11609	-11566	0.221	0.050	0.065	-0.12
parsopercularis thickness	0.568	-0.02	-14483	-14446	0.989	-0.01	0.739	-14482	-14438	0.653	-0.019	0.748	-0.02
parsorbitalis thickness	**0.024**	-0.08	-11735	-11698	0.372	-0.01	0.814	-11733	-11690	**0.035**	-0.087	0.449	-0.05
parstriangularis thickness	0.759	-0.01	-13813	-13776	0.817	-0.01	0.663	-13811	-13768	0.989	0.001	0.673	-0.03
pericalcarine thickness	0.536	-0.02	-12416	-12379	**0.008**	0.09	**0.008**	-12421	-12378	0.081	-0.072	0.116	0.10
postcentral thickness	0.211	-0.04	-15802	-15765	0.306	-0.06	0.061	-15804	-15761	0.669	0.018	**0.003**	-0.19
posteriorcingulate thickness	**0.034**	-0.07	-13588	-13551	0.733	-0.03	0.427	-13586	-13543	0.232	-0.049	0.104	-0.10
precentral thickness	0.547	-0.02	-16743	-16706	0.179	-0.06	0.061	-16745	-16702	0.533	0.026	0.043	-0.13
precuneus thickness	0.710	-0.01	-16940	-16903	0.223	0.04	0.235	-16939	-16896	0.242	-0.048	0.275	0.07

rostralanteriorcingulate thickness	***0.001***	-0.12	-11088	-11051	**0.015**	0.03	0.370	-11087	-11044	**0.007**	-0.111	0.058	-0.12
rostralmiddlefrontal thickness	***0.003***	-0.10	-15671	-15634	0.579	-0.03	0.315	-15670	-15627	**0.019**	-0.097	0.131	-0.09
superiorfrontal thickness	***$8.9 \cdot 10^{-5}$***	-0.13	-17795	-17758	0.056	0.0002	0.995	-17793	-17750	**0.009**	-0.107	**0.004**	-0.18
superiorparietal thickness	0.725	0.01	-17583	-17546	0.255	-0.04	0.268	-17582	-17539	0.473	0.030	0.519	-0.04
superiortemporal thickness	**0.049**	-0.07	-15790	-15753	0.439	-0.01	0.832	-15788	-15745	**0.040**	-0.085	0.474	-0.05
supramarginal thickness	0.980	0.00	-15627	-15590	0.578	-0.02	0.533	-15625	-15582	0.490	0.028	0.374	-0.06
temporalpole thickness	0.136	0.05	-9867	-9830	0.238	-0.02	0.604	-9865	-9822	0.119	0.064	0.614	0.03
transversetemporal thickness	0.364	0.03	-11161	-11124	0.812	0.01	0.815	-11159	-11116	0.922	0.004	0.169	0.09
Total average thickness	0.050	-0.07	-22896	-22859	0.835	-0.05	0.183	-22895	-22852	0.358	-0.038	**0.043**	-0.13

[1]Unadjusted *P* values are shown, with in **bold** those that are significant ($P < 0.05$) at the uncorrected level. Those that survived multiple testing correction (FDR < 0.05) are also indicated in *italic*

Supplementary Table 11. Age:diagnosis interaction effects for cortical surface area AIs. P-values and Cohen's *d* effect sizes for the main effects of diagnosis are shown for the primary and age:diagnosis interaction models, as well as for the stratification analyses separately in children and adults. P-values for the age:diagnosis interaction effects are also shown. Additionally, for both primary and interaction models the AIC and BIC model fit measures are shown..

	Primary analysis model				Age:diagnosis interaction model					Children only		Adults only	
**region**	**P_{diag}[1]**	***d***	**AIC**	**BIC**	**P_{diag}[1]**	***d***	**$P_{diag*age}$[1]**	**AIC**	**BIC**	**P_{diag}[1]**	***d***	**P_{diag}[1]**	***d***
bankssts surface area	0.332	0.03	-6952	-6915	0.365	-0.02	0.621	-6950	-6907	0.277	0.05	0.779	0.018
caudalanteriorcingulate surface area	0.662	-0.02	-5027	-4990	0.076	0.06	0.074	-5028	-4985	0.355	-0.04	0.542	0.038
caudalmiddlefrontal surface area	0.483	-0.02	-7903	-7866	0.571	-0.04	0.298	-7902	-7859	0.848	0.01	0.105	-0.102
cuneus surface area	0.215	-0.04	-8508	-8471	0.032	0.06	0.079	-8509	-8466	0.190	-0.05	0.609	-0.032
entorhinal surface area	0.108	-0.06	-4612	-4576	0.954	-0.03	0.406	-4611	-4568	0.230	-0.05	0.298	-0.066
frontalpole surface area	0.388	0.03	-6670	-6633	0.151	-0.04	0.245	-6669	-6626	0.230	0.05	0.917	-0.007
fusiform surface area	**0.010**	-0.09	-10454	-10417	0.157	0.01	0.860	-10452	-10409	**0.017**	-0.10	0.217	-0.077
inferiorparietal surface area	0.857	0.01	-10288	-10251	0.361	-0.03	0.345	-10286	-10243	0.687	0.02	0.836	-0.013
inferiortemporal surface area	0.585	-0.02	-9610	-9573	0.190	0.04	0.232	-9609	-9566	0.625	-0.02	0.739	-0.021
insula surface area	0.701	0.01	-11222	-11185	0.229	0.05	0.111	-11222	-11179	0.652	-0.02	0.185	0.083
isthmuscingulate surface area	0.611	0.02	-7974	-7937	0.089	-0.06	0.096	-7975	-7932	0.116	0.06	0.285	-0.067
lateraloccipital surface area	0.654	-0.02	-10709	-10672	0.247	0.04	0.282	-10708	-10665	0.197	-0.05	0.272	0.069
lateralorbitofrontal surface area	**0.001**	-0.11	-11771	-11734	0.042	0.02	0.622	-11769	-11726	***0.001***	-0.13	0.247	-0.073
lingual surface area	0.820	0.01	-9826	-9789	0.609	-0.02	0.646	-9824	-9781	0.597	0.02	0.734	-0.021
medialorbitofrontal surface area	0.002	0.11	-9475	-9438	**0.041**	-0.02	0.568	-9474	-9431	0.009	0.11	0.068	0.114
middletemporal surface area	0.661	-0.02	-11137	-11100	0.478	-0.04	0.290	-11136	-11093	0.919	0.00	0.385	-0.054
paracentral surface area	0.071	0.06	-9051	-9014	0.723	0.05	0.156	-9051	-9008	0.381	0.04	0.074	0.112
parahippocampal surface area	0.853	-0.01	-7631	-7594	0.181	0.05	0.154	-7631	-7588	0.457	-0.03	0.405	0.052
parsopercularis surface area	0.880	0.01	-7494	-7457	0.591	0.02	0.487	-7493	-7450	0.915	-0.004	0.670	0.027
parsorbitalis surface area	0.133	0.05	-9436	-9399	0.979	0.03	0.383	-9435	-9392	0.212	0.05	0.617	0.031
parstriangularis surface area	0.929	0.00	-7851	-7814	0.065	-0.07	**0.039**	-7853	-7810	0.516	0.03	0.267	-0.070
pericalcarine surface area	0.561	-0.02	-8905	-8868	0.790	-0.001	0.984	-8903	-8860	1.000	$1.1 \cdot 10^{-5}$	0.232	-0.075
postcentral surface area	0.430	0.03	-10895	-10858	0.463	-0.01	0.689	-10893	-10850	0.212	0.05	0.639	-0.029
posteriorcingulate surface area	0.475	-0.02	-7924	-7887	0.551	0.01	0.777	-7922	-7879	0.520	-0.03	0.698	-0.024
precentral surface area	0.154	-0.05	-12203	-12166	0.894	-0.03	0.344	-12202	-12159	0.528	-0.03	0.110	-0.100

precuneus surface area	0.915	-0.004	-11488	-11451	0.120	-0.06	0.066	-11489	-11446	0.474	0.03	0.215	-0.078
rostralanteriorcingulate surface area	0.317	-0.03	-6163	-6126	0.709	-0.005	0.895	-6161	-6118	0.974	0.00	0.056	-0.120
rostralmiddlefrontal surface area	0.694	0.01	-11642	-11605	0.628	-0.01	0.737	-11640	-11597	0.977	0.00	0.426	0.050
superiorfrontal surface area	0.569	0.02	-13194	-13157	0.090	-0.06	0.106	-13194	-13151	0.104	0.07	0.215	-0.078
superiorparietal surface area	0.688	-0.01	-11377	-11340	0.353	0.03	0.400	-11375	-11332	0.632	-0.02	0.984	0.001
superiortemporal surface area	0.339	-0.03	-11787	-11750	0.028	-0.11	**0.002**	-11795	-11752	0.327	0.04	**0.002**	-0.194
supramarginal surface area	0.954	0.002	-9191	-9154	0.159	-0.05	0.113	-9192	-9149	0.437	0.03	0.354	-0.058
temporalpole surface area	0.517	0.02	-7204	-7168	0.430	0.04	0.205	-7204	-7161	0.945	-0.003	0.273	0.069
transversetemporal surface area	0.870	0.01	-7600	-7563	0.407	-0.03	0.391	-7598	-7555	0.721	0.01	0.807	-0.015
totalsurf	0.344	-0.03	-21189	-21152	0.592	-0.04	0.253	-21188	-21145	0.966	0.002	0.149	-0.090

[1]Unadjusted *P* values are shown, with in **bold** those that are significant ($P < 0.05$) at the uncorrected level. Those that survived multiple testing correction (FDR < 0.05) are also indicated in *italic*

Supplementary Table 12. Age:diagnosis interaction effects for subcortical volume AIs. P-values and Cohen's *d* effect sizes for the main effects of diagnosis are shown for the primary and age:diagnosis interaction models, as well as for the stratification analyses separately in children and adults. P-values for the age:diagnosis interaction effects are also shown. Additionally, for both primary and interaction models the AIC and BIC model fit measures are shown..

	Primary analysis model				Age:diagnosis interaction model					Children only		Adults only	
**region**	**P_{diag}[1]**	***d***	**AIC**	**BIC**	**P_{diag}[1]**	***d***	**$P_{diag*age}$[1]**	**AIC**	**BIC**	**P_{diag}[1]**	***d***	**P_{diag}[1]**	***d***
Accumbens	0.425	-0.03	-7684	-7647	0.417	-0.05	0.169	-7684	-7641	0.990	0.001	0.180	-0.084
Amygdala	0.934	0.003	-9229	-9192	0.149	0.06	0.089	-9230	-9187	0.300	-0.04	0.109	0.101
CaudateNucleus	0.025	0.08	-12825	-12788	0.986	0.04	0.204	-12824	-12781	0.152	0.06	0.138	0.093
GlobusPallidus	0.593	0.02	-9466	-9429	0.056	0.09	**0.013**	-9470	-9427	0.818	-0.01	0.238	0.074
Hippocampus	0.485	-0.02	-11814	-11777	0.187	0.04	0.262	-11814	-11770	0.189	-0.05	0.572	0.035
LateralVentricle	0.741	-0.01	-3330	-3293	0.696	-0.02	0.529	-3329	-3286	0.892	-0.01	0.713	-0.024
Putamen	***0.001***	0.12	-13668	-13632	0.331	0.03	0.434	-13667	-13624	***0.001***	0.13	0.255	0.071
Thalamus	0.252	0.04	-13885	-13848	0.240	-0.02	0.480	-13884	-13840	0.400	0.03	0.609	0.032

[1]Unadjusted *P* values are shown, with in **bold** those that are significant ($P < 0.05$) at the uncorrected level. Those that survived multiple testing correction (FDR < 0.05) are also indicated in *italic*

Supplementary Table 13. Association of IQ with asymmetry within cases (top) and within controls (bottom), for the AIs that showed significant effects of diagnosis in the primary analysis.

		β-value			Standard Error			*t*-value			*P*-value[1]		
**AI region**	**N total**	**IQ**	**sex**	**age**	**IQ**	**sex**	**age**	**IQ**	**sex**	**age**	**IQ**	**sex**	**age**
**within cases:**													
fusiform thickness	1360	-0.00007	0.00054	-0.00001	0.00004	0.00198	0.00011	-1.75	0.27	-0.13	0.081	0.784	0.897
inferiortemporal thickness	1361	-0.00008	-0.00124	0.00004	0.00005	0.00230	0.00012	-1.72	-0.54	0.36	0.085	0.591	0.718
isthmuscingulate thickness	1358	-0.00007	0.00557	0.00038	0.00006	0.00304	0.00013	-1.25	1.83	2.88	0.212	0.067	**0.004**
medialorbitofrontal thickness	1360	-0.00004	0.00073	0.00046	0.00005	0.00272	0.00014	-0.68	0.27	3.21	0.498	0.789	**0.001**
rostralanteriorcingulate thickness	1357	0.00019	0.00950	0.00004	0.00008	0.00376	0.00020	2.49	2.53	0.20	**0.013**	**0.012**	0.844
rostralmiddlefrontal thickness	1362	-0.00003	0.00245	-0.00031	0.00004	0.00187	0.00011	-0.75	1.31	-2.89	0.456	0.190	**0.004**
superiorfrontal thickness	1362	-0.00003	-0.00001	0.00005	0.00003	0.00144	0.00008	-0.91	-0.01	0.56	0.365	0.996	0.577
lateralorbitofrontal surface area	1351	-0.00001	0.00286	0.00003	0.00007	0.00343	0.00018	-0.21	0.83	0.19	0.833	0.404	0.847
medialorbitofrontal surface area	1352	-0.00006	0.00055	0.00009	0.00010	0.00478	0.00024	-0.66	0.11	0.38	0.509	0.909	0.702
Putamen	1372	0.00003	0.00424	0.00052	0.00006	0.00278	0.00014	0.58	1.52	3.68	0.563	0.128	**0.0002**
**within controls:**													
fusiform thickness	1432	-0.00002	0.00313	0.00009	0.00005	0.00162	0.00011	-0.42	1.94	0.84	0.677	0.053	0.404
inferiortemporal thickness	1431	-0.00010	0.00262	0.00008	0.00006	0.00187	0.00013	-1.82	1.40	0.66	0.069	0.161	0.511
isthmuscingulate thickness	1431	0.00001	0.00241	0.00026	0.00008	0.00261	0.00015	0.13	0.92	1.74	0.896	0.358	0.082
medialorbitofrontal thickness	1431	0.00003	-0.00183	0.00036	0.00007	0.00247	0.00017	0.39	-0.74	2.12	0.695	0.458	**0.034**
rostralanteriorcingulate thickness	1428	0.00009	-0.00262	-0.00020	0.00010	0.00321	0.00022	0.91	-0.82	-0.93	0.361	0.415	0.353
rostralmiddlefrontal thickness	1431	-0.00008	0.00133	-0.00013	0.00005	0.00170	0.00013	-1.60	0.78	-1.04	0.111	0.434	0.299
superiorfrontal thickness	1432	-0.00012	-0.00140	-0.00002	0.00004	0.00118	0.00009	-3.41	-1.18	-0.20	**0.001**	0.236	0.839
lateralorbitofrontal surface area	1426	-0.00008	-0.00191	-0.00026	0.00008	0.00282	0.00018	-0.96	-0.68	-1.45	0.335	0.498	0.147
medialorbitofrontal surface area	1426	-0.00018	-0.00285	0.00029	0.00012	0.00406	0.00026	-1.51	-0.70	1.09	0.132	0.482	0.276
Putamen	1426	0.00005	0.00145	0.00024	0.00006	0.00206	0.00015	0.82	0.71	1.67	0.414	0.480	0.094

[1]Unadjusted *P* values are shown, with in **bold** those that are significant ($P < 0.05$) at the uncorrected level.

Supplementary Table 14. Association of log10-normalized ADOS severity scores with brain asymmetry within cases, for the AIs that showed significant effects of diagnosis in the primary analysis.

		β-value			Standard Error			*t*-value			*P*-value[1]		
**AI region**	**N cases**	**ADOS**	**sex**	**age**	**ADOS**	**sex**	**age**	**ADOS**	**sex**	**age**	**ADOS**	**sex**	**age**
fusiform thickness	855	0.0059	0.0036	-0.0001	0.0060	0.0031	0.0001	0.98	1.17	-0.82	0.325	0.242	0.415
inferiortemporal thickness	857	-0.0016	0.0007	$3.37 \cdot 10^{-5}$	0.0067	0.0034	0.0001	-0.24	0.20	0.25	0.807	0.841	0.805
isthmuscingulate thickness	853	0.0192	0.0040	0.0006	0.0069	0.0046	0.0001	2.76	0.87	3.97	**0.006**	0.382	**$7.8 \cdot 10^{-5}$**
medialorbitofrontal thickness	856	0.0047	0.0002	0.0003	0.0078	0.0042	0.0002	0.61	0.04	2.22	0.543	0.970	**0.027**
rostralanteriorcingulate thickness	854	-0.0072	0.0065	$1.93 \cdot 10^{-5}$	0.0117	0.0060	0.0002	-0.61	1.07	0.08	0.539	0.284	0.936
rostralmiddlefrontal thickness	858	0.0015	0.0024	-0.0004	0.0059	0.0029	0.0001	0.26	0.84	-2.86	0.796	0.401	**0.004**
superiorfrontal thickness	858	-0.0009	-0.0001	$-5.32 \cdot 10^{-6}$	0.0043	0.0021	0.0001	-0.20	-0.07	-0.06	0.842	0.948	0.953
lateralorbitofrontal surface area	852	0.0039	0.0061	0.0001	0.0099	0.0050	0.0002	0.39	1.22	0.67	0.696	0.223	0.503
medialorbitofrontal surface area	854	-0.0032	0.0009	0.0002	0.0124	0.0066	0.0002	-0.26	0.13	0.84	0.797	0.894	0.401
Putamen	866	0.0077	0.0036	0.0004	0.0067	0.0035	0.0001	1.15	1.03	3.21	0.252	0.303	**0.001**

[1]Unadjusted *P* values are shown, with in **bold** those that are significant ($P < 0.05$) at the uncorrected level.

Supplementary Table 15 Association of medication use with brain asymmetry within cases, for the AIs that showed significant effects of diagnosis in the primary analysis.

AI region	N cases	β-value			Standard Error			*t*-value			*P*-value[1]		
		med	sex	age	med	sex	age	med	sex	age	med	sex	age
fusiform thickness	804	-0.0023	0.0046	0.0001	0.0023	0.0029	0.0002	-1.00	1.60	0.77	0.316	0.110	0.444
inferiortemporal thickness	804	-0.0028	0.0022	0.0002	0.0026	0.0032	0.0002	-1.08	0.69	0.94	0.280	0.493	0.349
isthmuscingulate thickness	799	0.0031	-0.0013	0.0007	0.0035	0.0045	0.0002	0.88	-0.29	4.10	0.377	0.775	**$4.6 \cdot 10^{-5}$**
medialorbitofrontal thickness	805	0.0000	0.0008	0.0004	0.0031	0.0039	0.0002	0.00	0.21	2.05	0.997	0.837	**0.041**
rostral anterior cingulate thickness	803	0.0045	0.0077	-0.0001	0.0044	0.0054	0.0003	1.03	1.41	-0.36	0.302	0.159	0.718
rostralmiddlefrontal thickness	807	0.0018	0.0028	-0.0003	0.0021	0.0027	0.0002	0.84	1.05	-1.96	0.400	0.295	0.051
superiorfrontal thickness	807	0.0006	-0.0005	0.0000	0.0017	0.0021	0.0001	0.35	-0.24	-0.36	0.730	0.814	0.717
lateralorbitofrontal surface area	801	0.0030	0.0032	0.0001	0.0036	0.0045	0.0003	0.84	0.71	0.54	0.401	0.477	0.592
medialorbitofrontal surface area	803	0.0021	-0.0055	$4.4 \cdot 10^{-5}$	0.0048	0.0060	0.0003	0.43	-0.91	0.14	0.668	0.361	0.889
Putamen	813	0.0018	-0.0015	0.0005	0.0027	0.0034	0.0002	0.67	-0.43	2.71	0.502	0.666	**0.007**

[1]Unadjusted *P* values are shown, with in **bold** those that are significant ($P < 0.05$) at the uncorrected level.

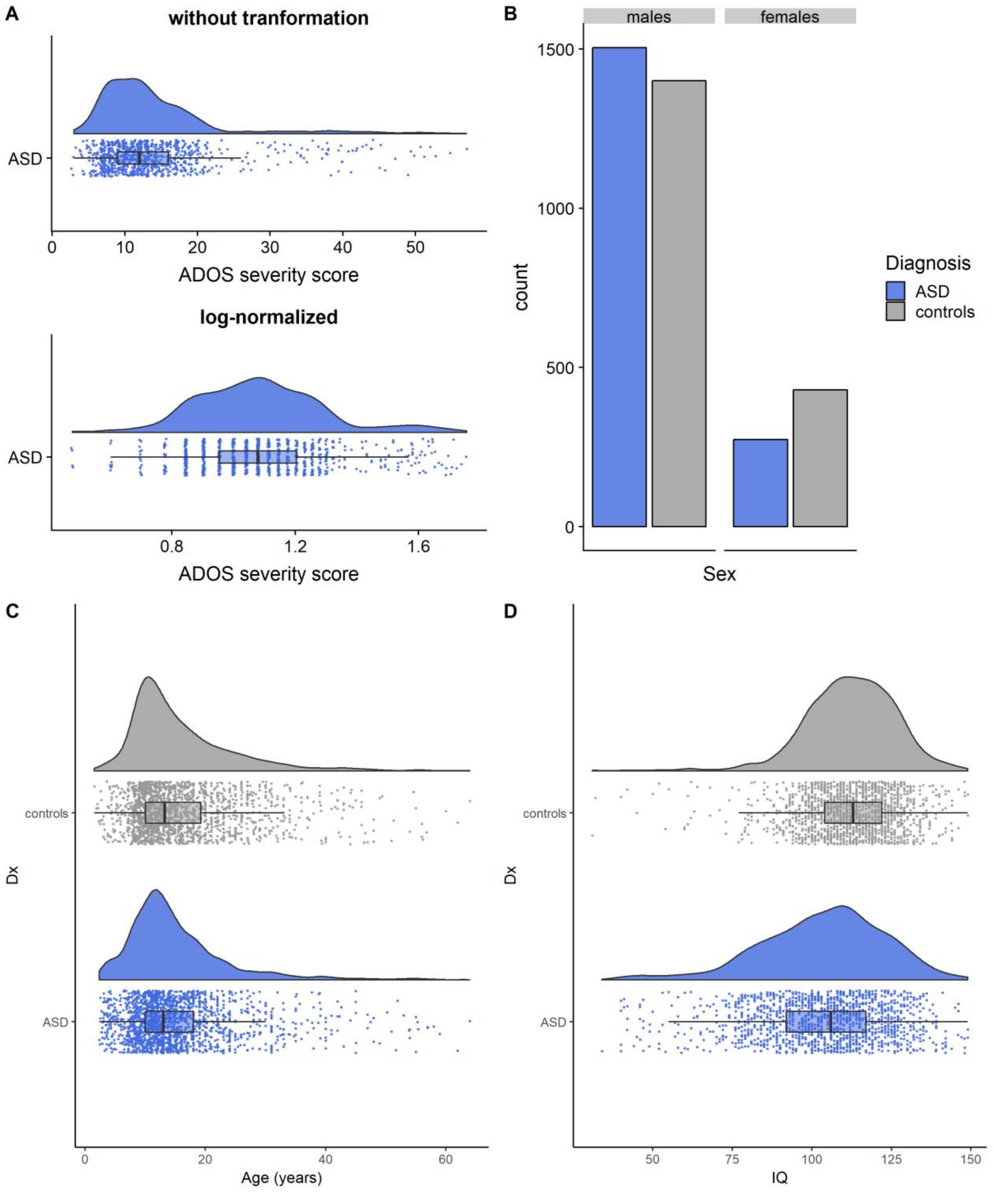

Supplementary Figure 1. Distributions of variables of interest. (A) ADOS severity scores within cases, (B) sex, (C) age, (D) IQ. ADOS severity scores did not follow a normal distribution (top), so data were transformed using $\log_{10}$ (bottom). *Blue* = ASD group; *grey* = control group.

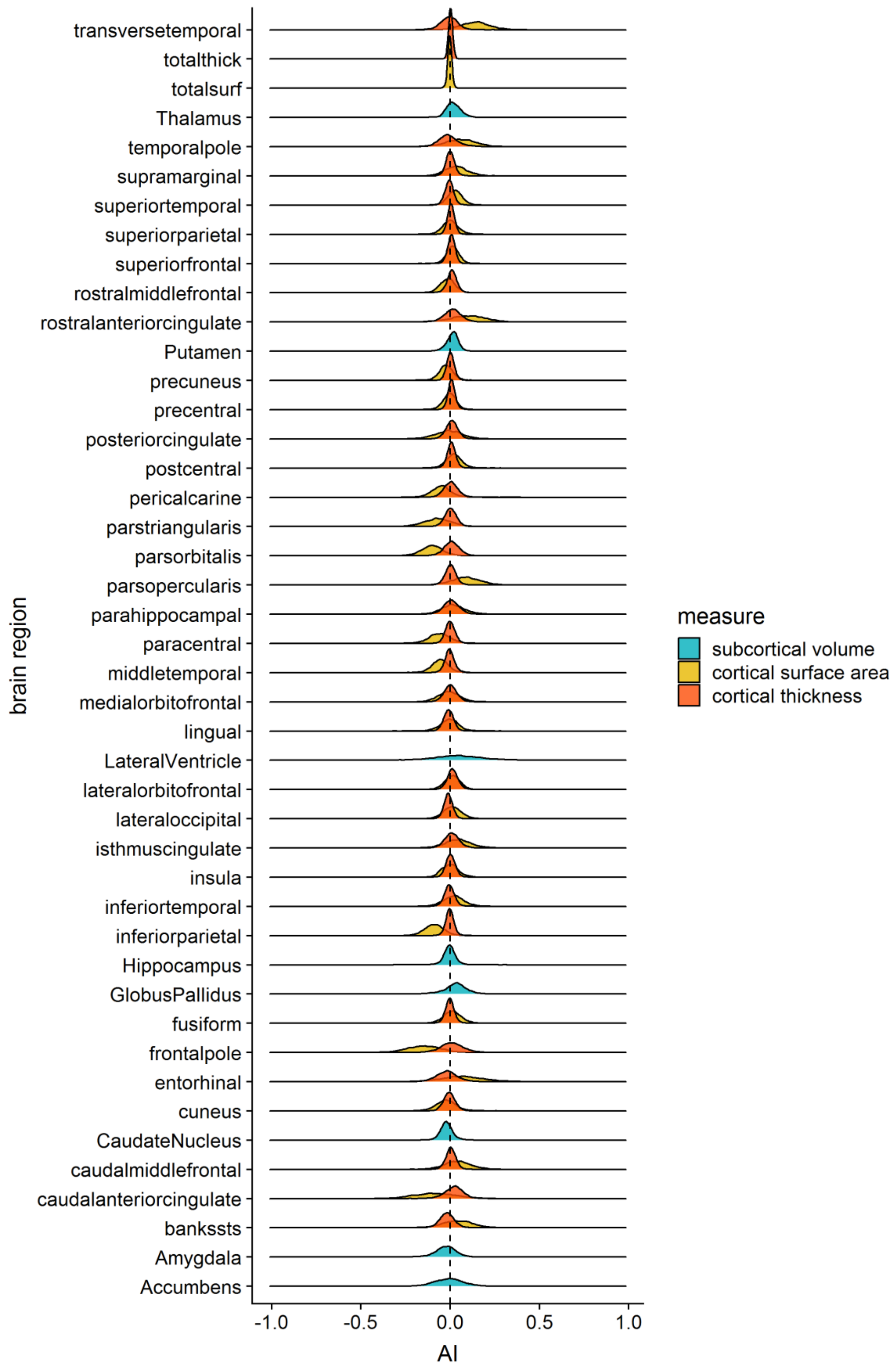

Supplementary Figure 2. Joyplot of the distributions of AIs in the total sample (without winsorization). Shown for subcortical volumes (*cyan*), cortical surface areas (*orange*), and cortical thicknesses (*red*). Lateral ventricles were categorized among subcortical measures

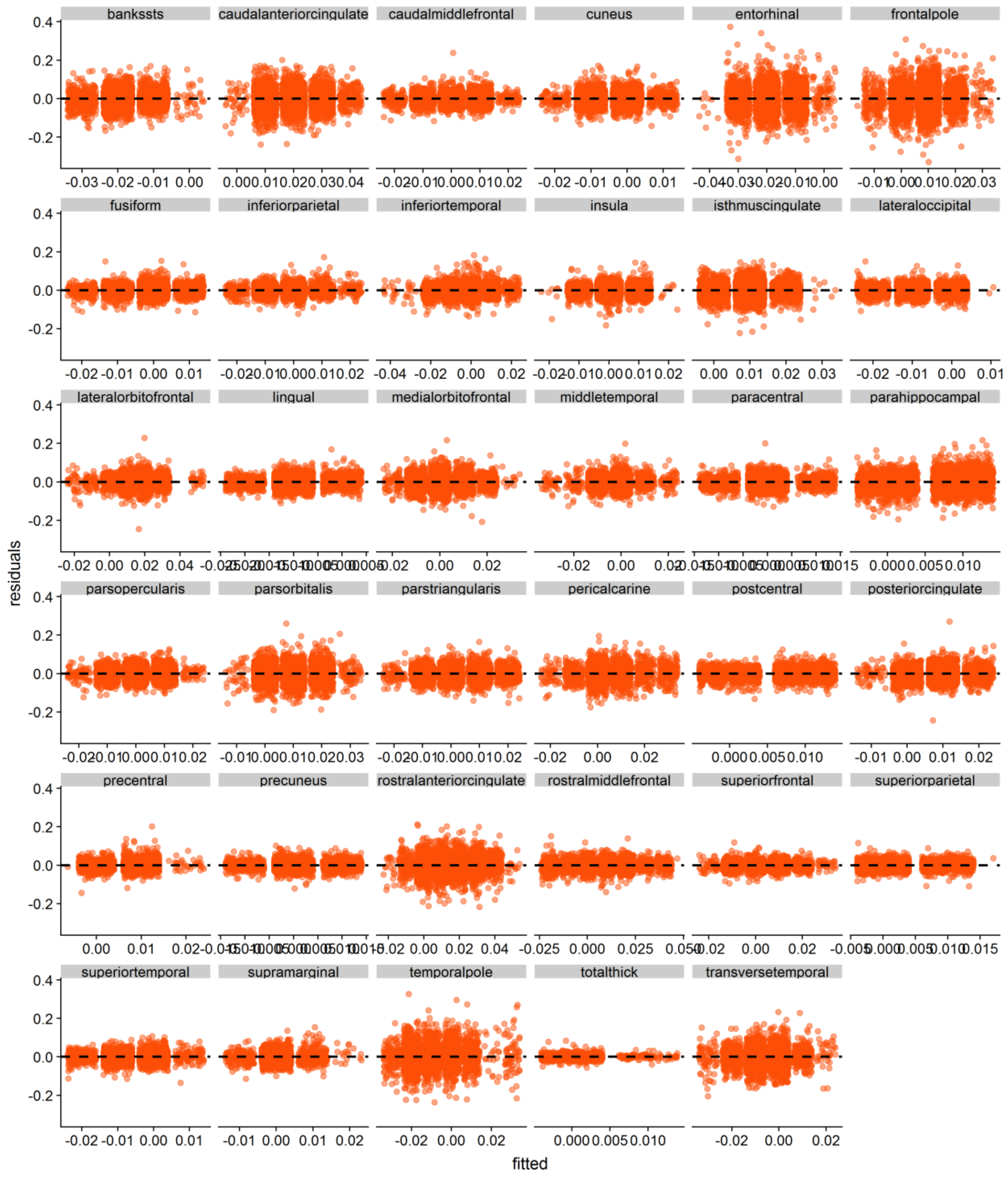

Supplementary Figure 3. Residual plots of the linear mixed effects model analysis of cortical thickness AIs and the AI of the total average cortical thickness (totalthick).

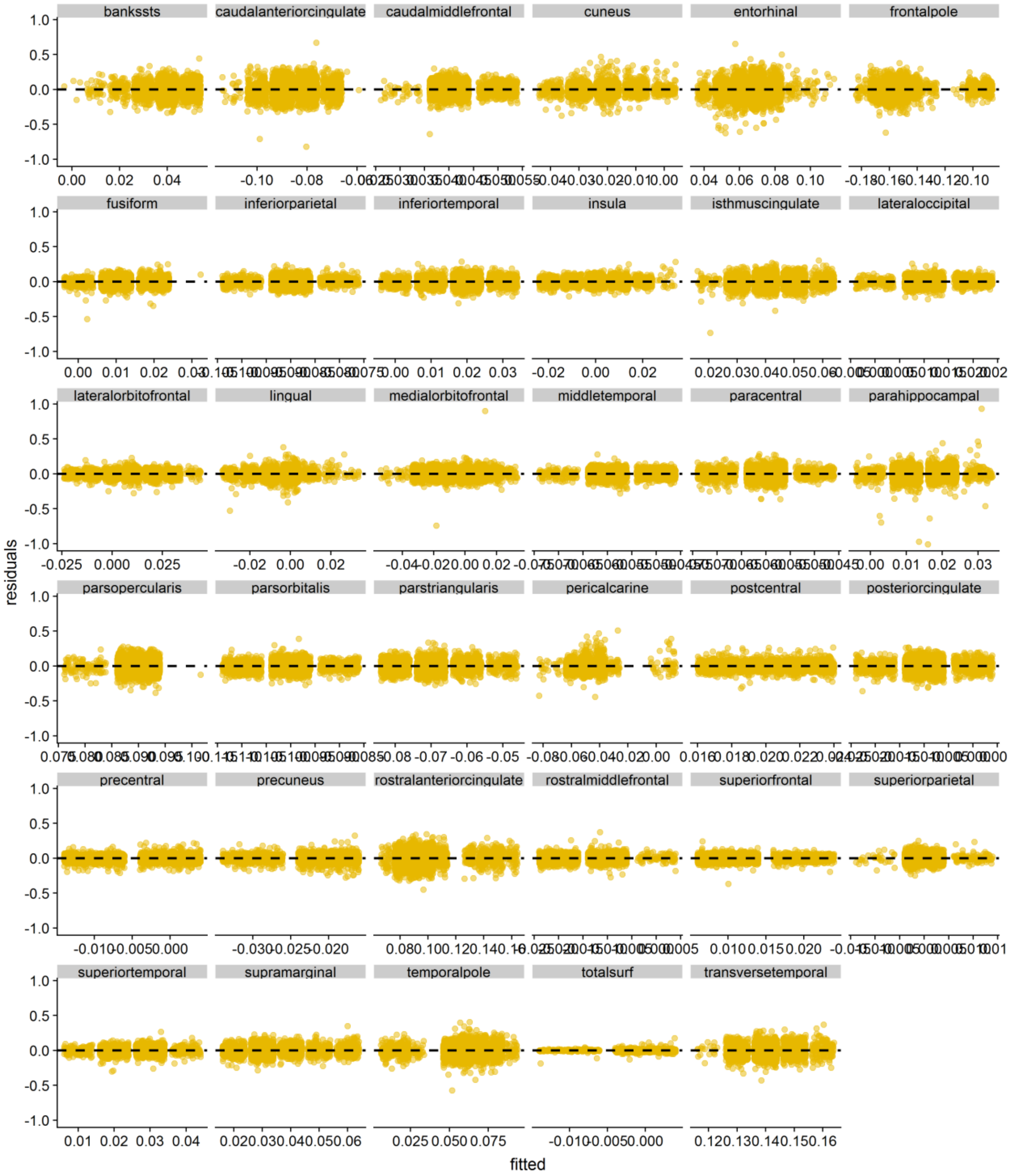

Supplementary Figure 4. Residual plots of the linear mixed effects model analysis of cortical surface area AIs and the AI of the total cortical surface area (totalsurf).

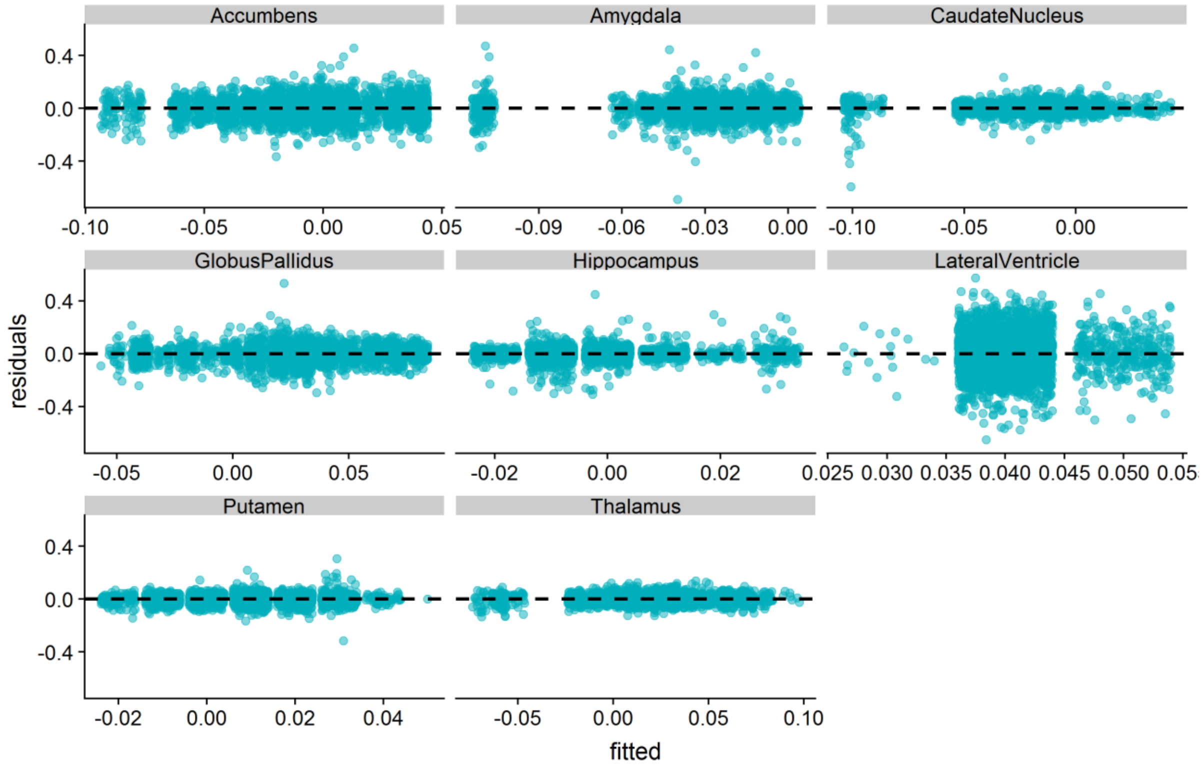

Supplementary Figure 5. Residual plots of the linear mixed effects model analysis of subcortical volume AIs and the AI of the lateral ventricles.

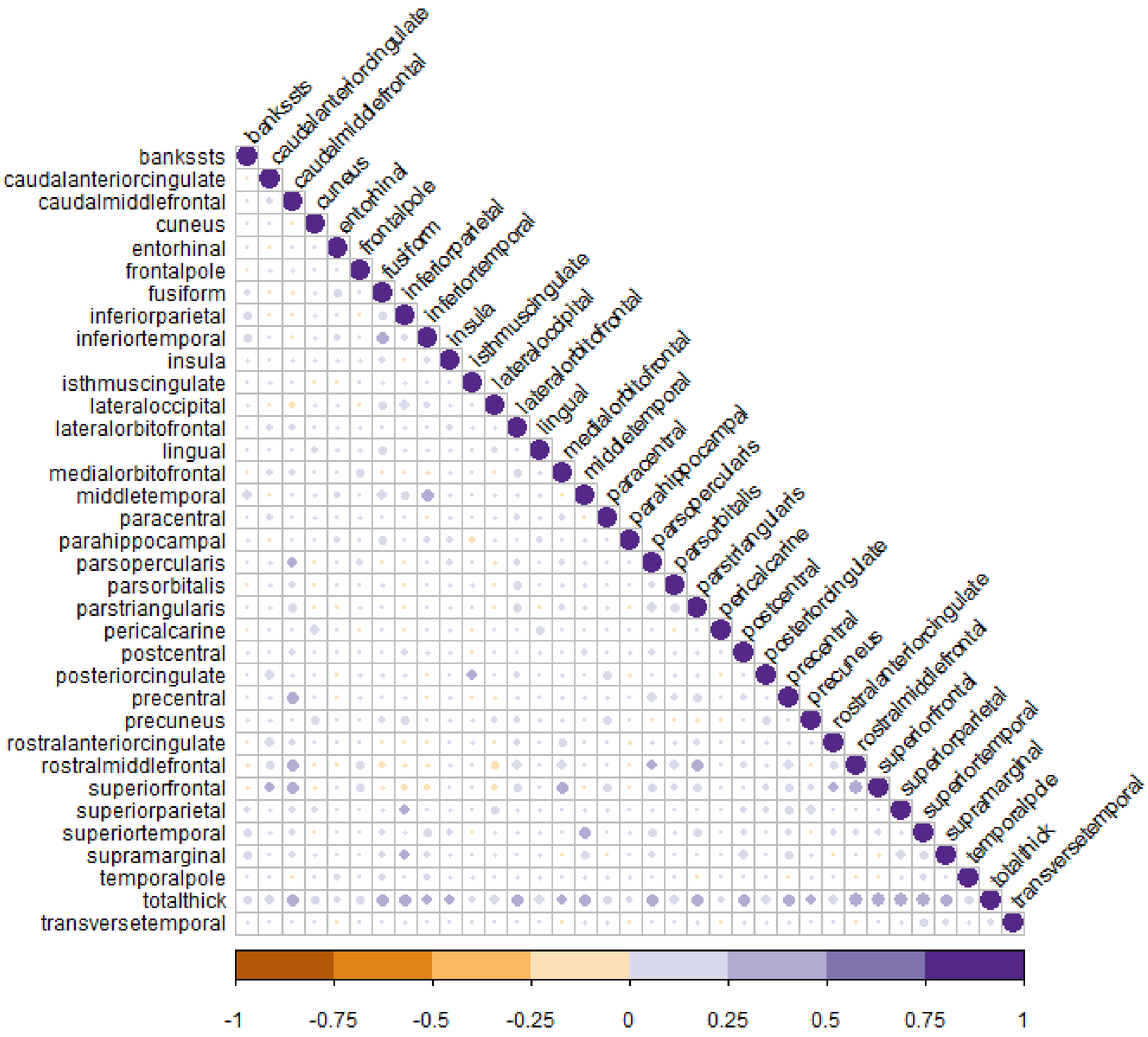

Supplementary Figure 6. Correlations between AIs of cortical thickness. Correlations ranged from -015 to 0.47. Negative correlations are shown in *orange*, and positive correlations are shown in *purple*. Color intensities and circle sizes are proportional to the magnitudes of the correlation coefficients.

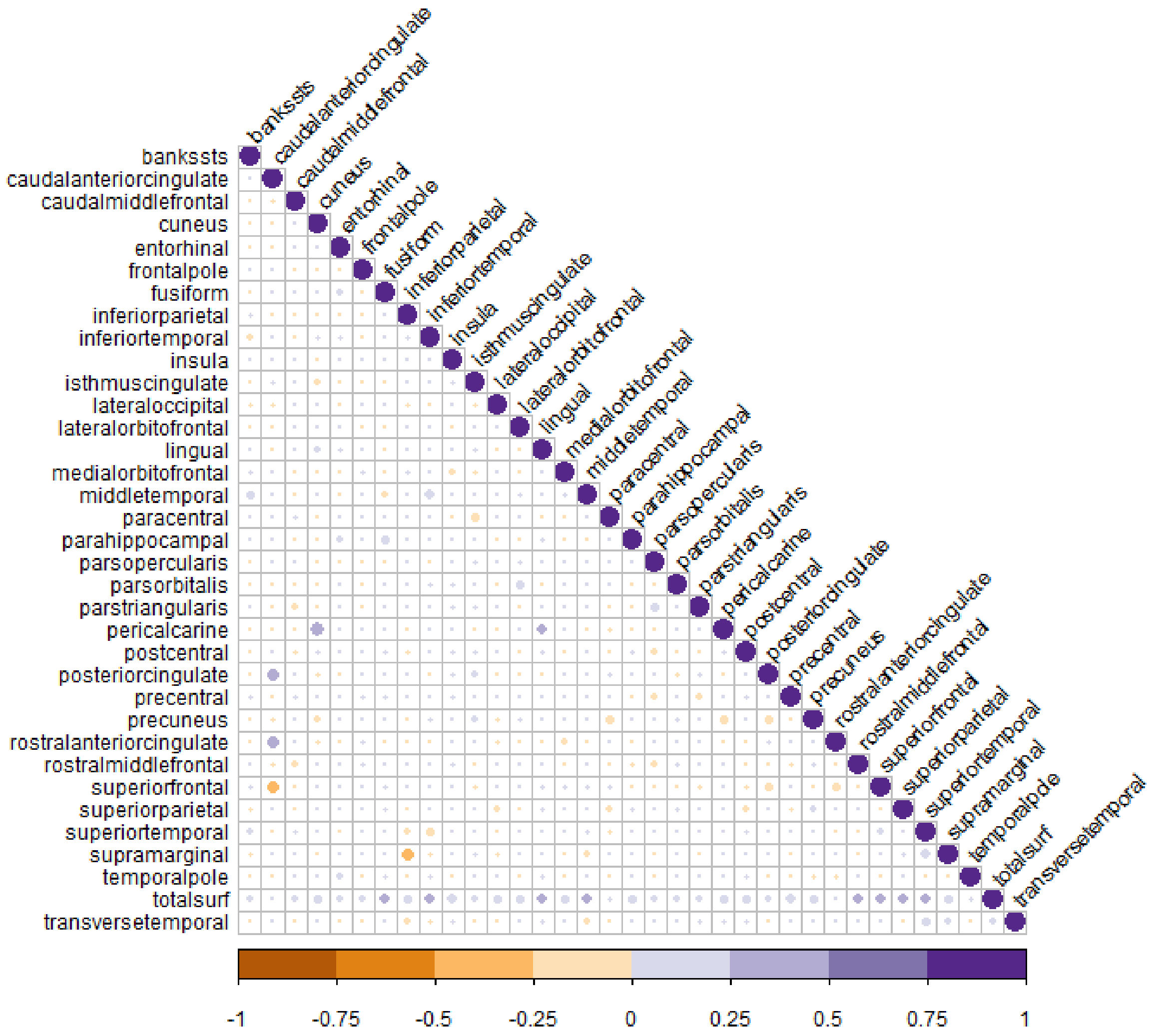

Supplementary Figure 7. Correlations between AIs of cortical surface areas. Correlations ranged from -035 to 0.49. Negative correlations are shown in *orange*, and positive correlations are shown in *purple*. Color intensities and circle sizes are proportional to the magnitudes of the correlation coefficients.

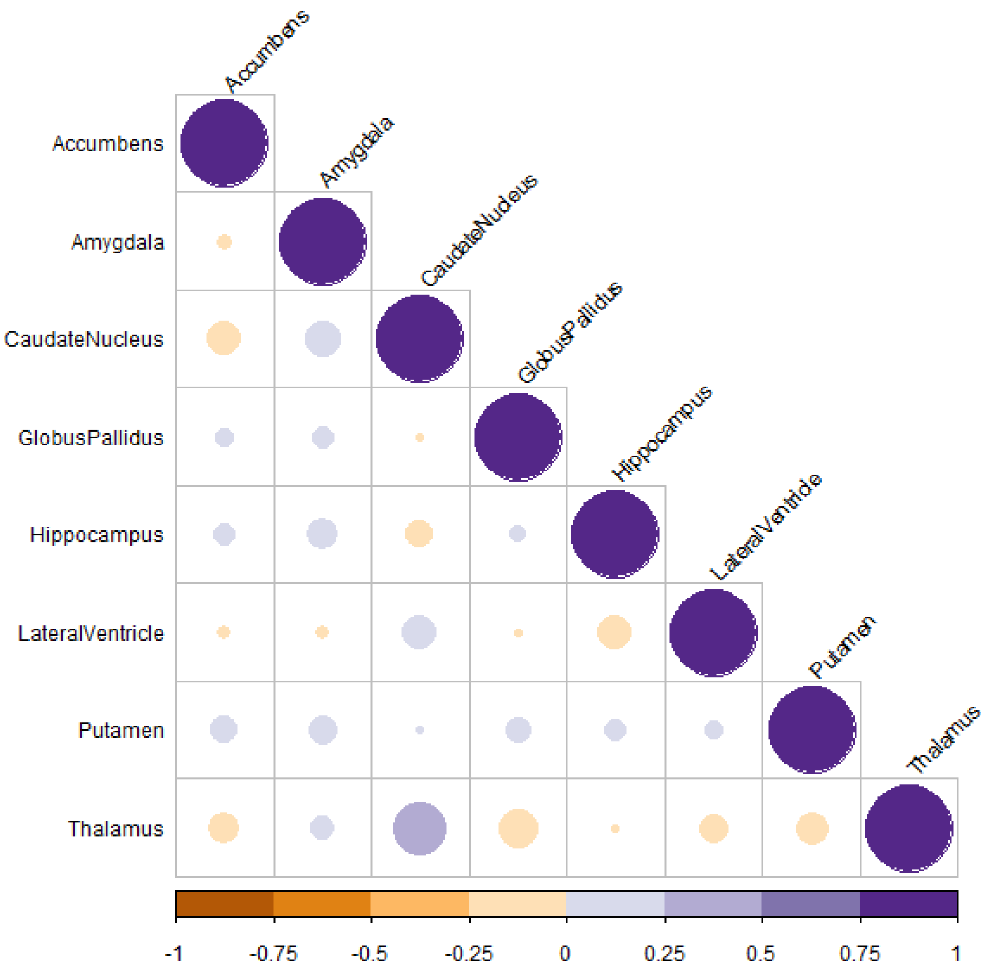

Supplementary Figure 8. Correlations between AIs of subcortical volumes. Correlations ranged from -020 to 0.38. Negative correlations are shown in *orange*, and positive correlations are shown in *purple*. Color intensities and circle sizes are proportional to the magnitudes of the correlation coefficients

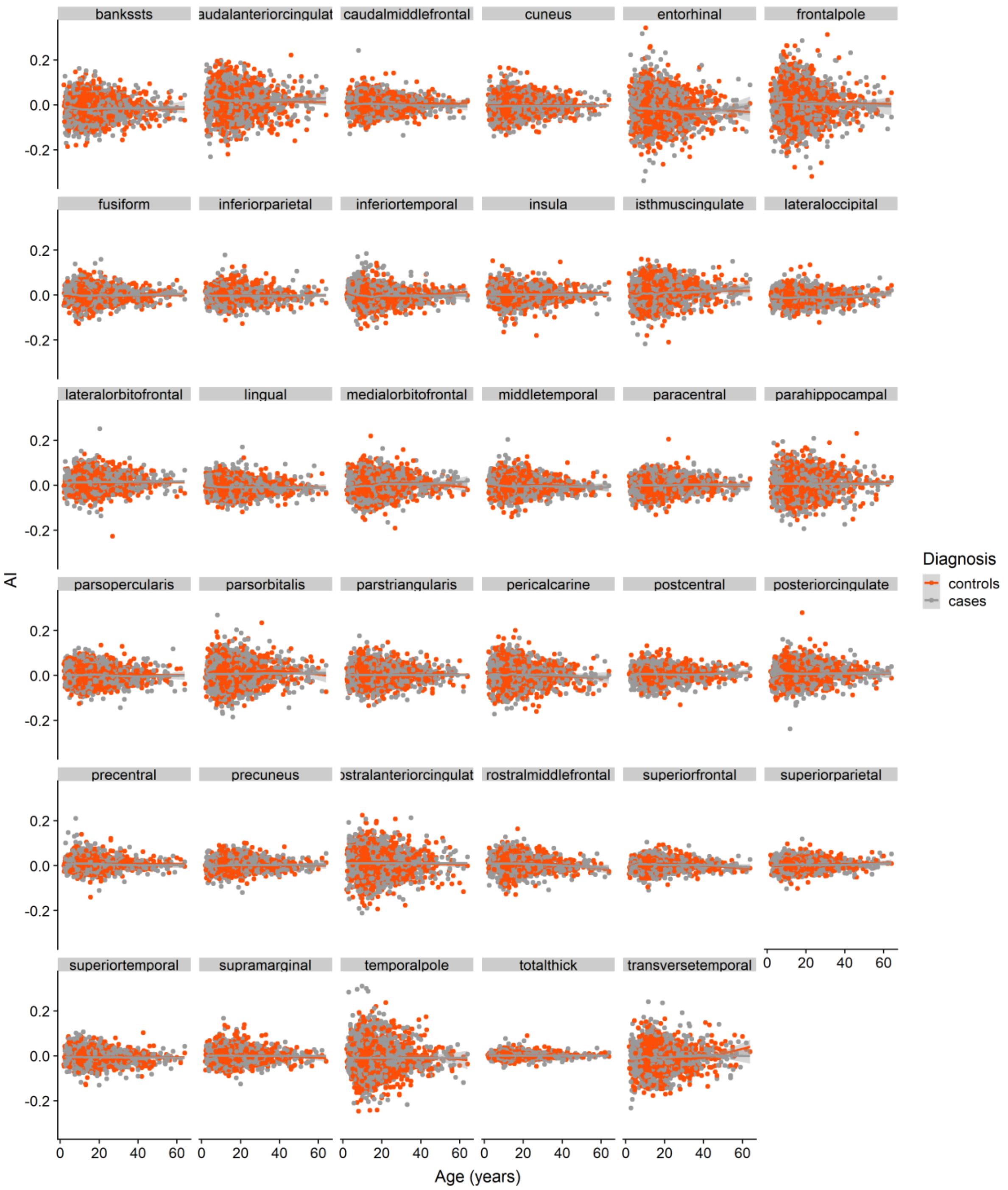

Supplementary Figure 9. Scatter plots of the relationships between age and AIs of the total and regional cortical thicknesses.

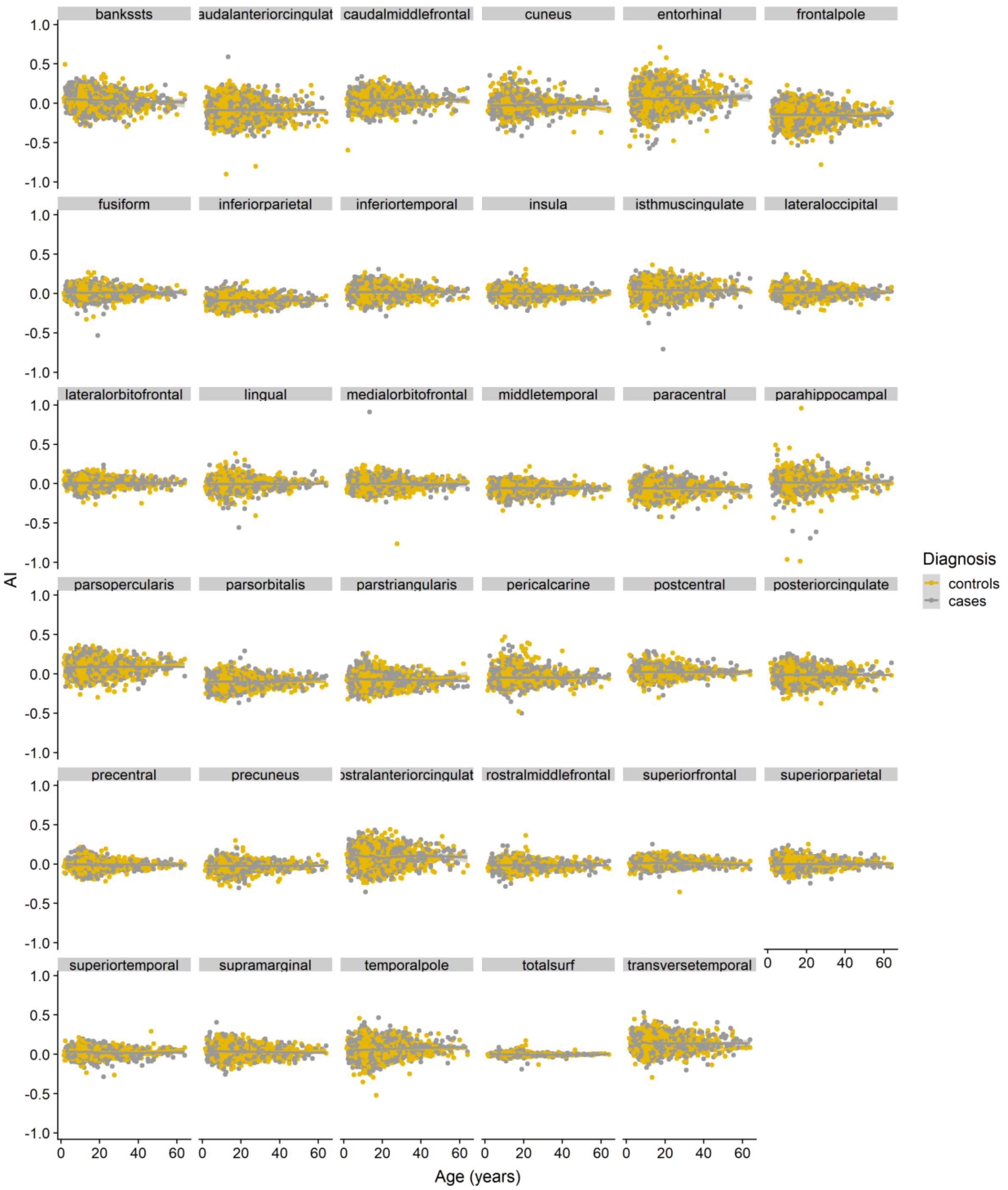

Supplementary Figure 10. Scatter plots of the relationships between age and AIs of the total and regional cortical surface areas.

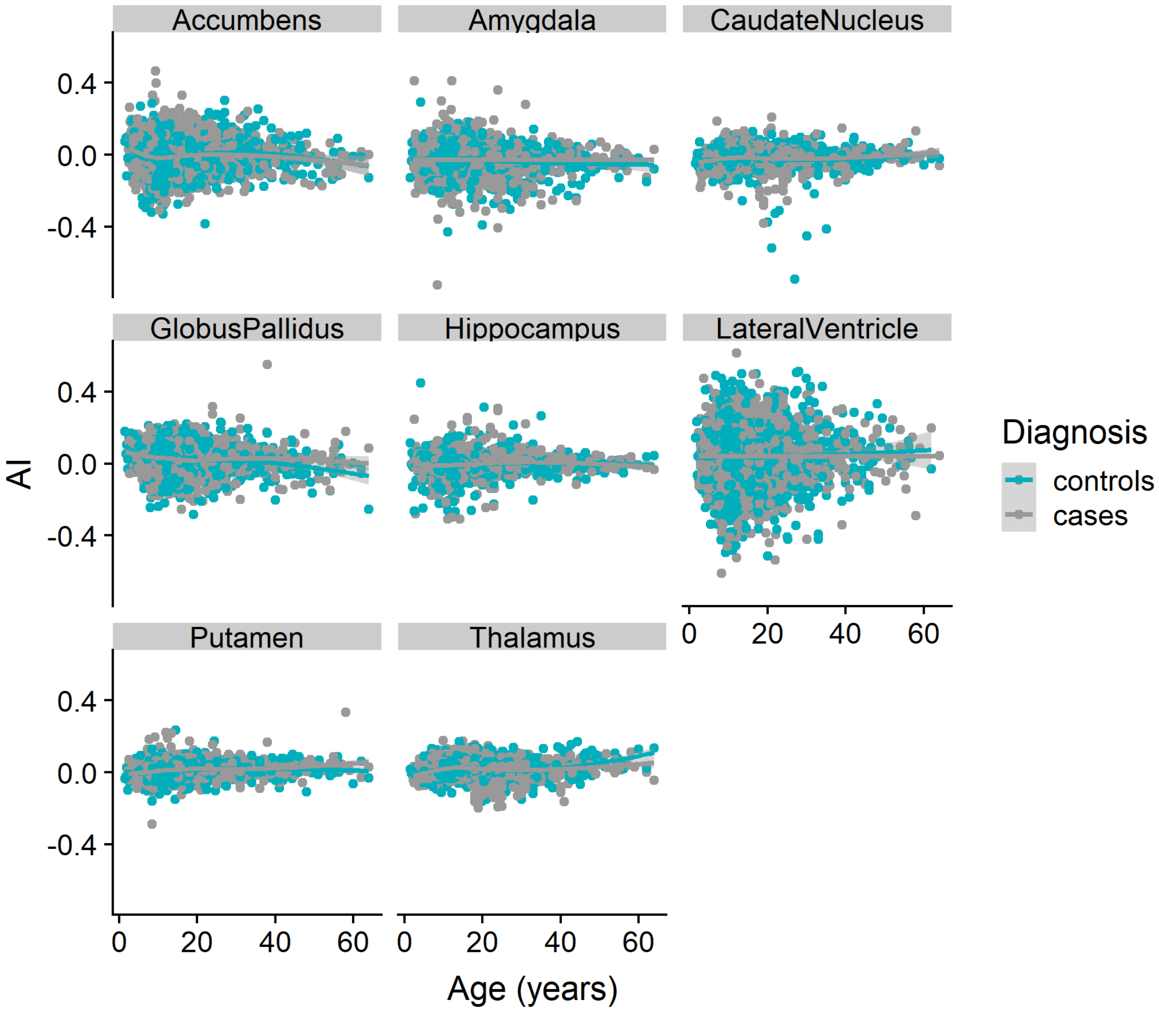

Supplementary Figure 11. Scatter plots of the relationships between age and AIs of subcortical volumes and lateral ventricles.
