# Supplementary material for: Altered structural brain asymmetry in autism spectrum disorder in a study of 54 datasets
Source: Nat Commun. 2019 Oct 31;10:4958. doi: 10.1038/s41467-019-13005-8 (PMC6823355; doi:10.1038/s41467-019-13005-8)
Supplement: Supplementary file 3 — Description of Additional Supplementary Files [file 41467_2019_13005_MOESM3_ESM.pdf]

Description of Additional Supplementary Files

File Name: Supplementary Software 1
Description: Randomized demo data, R scripts, and a README file written according to the *Nature Communications* software policy. Please note that four R scripts are included, but that the demo_SCRIPT2RUN.R file is the only script that needs to be launched (it automatically runs the other scripts in the folder).
