# Supplementary material for: Altered structural brain asymmetry in autism spectrum disorder in a study of 54 datasets
Source: Nat Commun. 2019 Oct 31;10:4958. doi: 10.1038/s41467-019-13005-8 (PMC6823355; doi:10.1038/s41467-019-13005-8)
Supplement: Supplementary file 5 — Reporting Summary [file 41467_2019_13005_MOESM5_ESM.pdf]

nature research

Corresponding author(s): Clyde Francks

Last updated by author(s): 30-08-2019

# Reporting Summary

Nature Research wishes to improve the reproducibility of the work that we publish. This form provides structure for consistency and transparency in reporting. For further information on Nature Research policies, see Authors & Referees and the Editorial Policy Checklist .

## Statistics

For all statistical analyses, confirm that the following items are present in the figure legend, table legend, main text, or Methods section.

| n/a | Confirmed | |
|---|---|---|
| ☐ | ☒ | The exact sample size (*n*) for each experimental group/condition, given as a discrete number and unit of measurement |
| ☐ | ☒ | A statement on whether measurements were taken from distinct samples or whether the same sample was measured repeatedly |
| ☐ | ☒ | The statistical test(s) used AND whether they are one- or two-sided<br>*Only common tests should be described solely by name; describe more complex techniques in the Methods section.* |
| ☐ | ☒ | A description of all covariates tested |
| ☐ | ☒ | A description of any assumptions or corrections, such as tests of normality and adjustment for multiple comparisons |
| ☐ | ☒ | A full description of the statistical parameters including central tendency (e.g. means) or other basic estimates (e.g. regression coefficient) AND variation (e.g. standard deviation) or associated estimates of uncertainty (e.g. confidence intervals) |
| ☐ | ☒ | For null hypothesis testing, the test statistic (e.g. *F*, *t*, *r*) with confidence intervals, effect sizes, degrees of freedom and *P* value noted<br>*Give P values as exact values whenever suitable.* |
| ☒ | ☐ | For Bayesian analysis, information on the choice of priors and Markov chain Monte Carlo settings |
| ☒ | ☐ | For hierarchical and complex designs, identification of the appropriate level for tests and full reporting of outcomes |
| ☐ | ☒ | Estimates of effect sizes (e.g. Cohen's *d*, Pearson's *r*), indicating how they were calculated |

*Our web collection on statistics for biologists contains articles on many of the points above.*

## Software and code

Policy information about availability of computer code

| | |
|---|---|
| Data collection | MRI scanning of 54 datasets (see supplementary information for scanner manufacturers and field strengths). FreeSurfer software's default process (version 5.1 or 5.3, specified in supplementary information per dataset) was used for data collection from brain MRI data. |
| Data analysis | R (version 3.3.3) was used for data analysis |

For manuscripts utilizing custom algorithms or software that are central to the research but not yet described in published literature, software must be made available to editors/reviewers. We strongly encourage code deposition in a community repository (e.g. GitHub). See the Nature Research guidelines for submitting code & software for further information.

## Data

Policy information about availability of data

All manuscripts must include a data availability statement. This statement should provide the following information, where applicable:

- Accession codes, unique identifiers, or web links for publicly available datasets
- A list of figures that have associated raw data
- A description of any restrictions on data availability

Requests to access the data used in the current study will be considered in relation to the relevant consents, rules and regulations applying to each dataset, and can be made via the corresponding author.

# Field-specific reporting

Please select the one below that is the best fit for your research. If you are not sure, read the appropriate sections before making your selection.

☒

# Life sciences study design

All studies must disclose on these points even when the disclosure is negative.

| | |
|---|---|
| Sample size | Exact sample sizes for each model were determined in R, where the number of rows within the data equals the number of subjects. This was the largest study of brain asymmetry in autism spectrum disorder to have been performed, by an order of magnitude. The paper includes indicative power analyses to show the minimum effect sizes possible to detect with 80% power, given the sample sizes. |
| Data exclusions | Three datasets comprising either cases only, or controls only, were removed in this study, as our analysis model included random intercepts for 'dataset', and diagnosis was fully confounded with dataset for these three. Quality control and outlier removal is described in the paper. Analysis was repeated before and after outlier exclusion. |
| Replication | No replication study was possible. We used all available case-control datasets in discovery analysis in order maximise power. |
| Randomization | Not relevant to our study, because of the case-control design |
| Blinding | See above |

# Reporting for specific materials, systems and methods

We require information from authors about some types of materials, experimental systems and methods used in many studies. Here, indicate whether each material, system or method listed is relevant to your study. If you are not sure if a list item applies to your research, read the appropriate section before selecting a response.

## Materials & experimental systems

| n/a | Involved in the study | |
|---|---|---|
| ☒ | ☐ | Antibodies |
| ☒ | ☐ | Eukaryotic cell lines |
| ☒ | ☐ | Palaeontology |
| ☒ | ☐ | Animals and other organisms |
| ☐ | ☒ | Human research participants |
| ☐ | ☒ | Clinical data |

## Methods

| n/a | Involved in the study | |
|---|---|---|
| ☒ | ☐ | ChIP-seq |
| ☒ | ☐ | Flow cytometry |
| ☐ | ☒ | MRI-based neuroimaging |

## Human research participants

Policy information about studies involving human research participants

| | |
|---|---|
| Population characteristics | The 54 datasets used in our study comprised 1,778 people with ASD (N = 1,504 males; median age = 13 years; range = 2 to 64 years) and 1,829 typically developing controls (N = 1,400 males; median age = 13 years; range = 2 to 64 years). Information on sex, ADOS severity and IQ are in the manuscript. |
| Recruitment | The study used data from previously published studies where details are described. |
| Ethics oversight | The 54 datasets were all recruited and assessed following approval by the appropriate ethics boards in the relevant countries |

Note that full information on the approval of the study protocol must also be provided in the manuscript.

## Clinical data

Policy information about clinical studies

All manuscripts should comply with the ICMJEguidelines for publication of clinical research and a completedCONSORT checklist must be included with all submissions.

| | |
|---|---|
| Clinical trial registration | *Provide the trial registration number from ClinicalTrials.gov or an equivalent agency.* |
| Study protocol | *Note where the full trial protocol can be accessed OR if not available, explain why.* |
| Data collection | This observational study used case-control data from previously published studies, where details are described. |
| Outcomes | *Describe how you pre-defined primary and secondary outcome measures and how you assessed these measures.* |

# Magnetic resonance imaging

## Experimental design

| | |
|---|---|
| Design type | structural MRI, T1 images |
| Design specifications | Structural T1-weighted brain MRI scans were acquired at each study site. Images were acquired using different field strengths (1.5 T or 3 T) and scanner types, as specified per dataset in supplementary information. |
| Behavioral performance measures | Not applicable |

## Acquisition

| | |
|---|---|
| Imaging type(s) | structural |
| Field strength | 1.5T or 3T |
| Sequence & imaging parameters | 54 separate datasets |
| Area of acquisition | Cortical parcellations and subcortical segmentations were performed with the freely available and validated software FreeSurfer (versions 5.1 or 5.3), using the default 'recon-all' pipeline.<br>The data used in the current study were thickness and surface area measures for each of 34 bilaterally paired cortical regions, the latter as defined with the Desikan-Killiany atlas, as well as the average cortical thickness and total surface area per entire hemisphere. In addition, left and right volumes of seven bilaterally paired subcortical structures, plus the lateral ventricles, were analyzed. |

Diffusion MRI ☐ Used ☒ Not used

## Preprocessing

| | |
|---|---|
| Preprocessing software | FreeSurfer version 5.1 or 5.3 (as specified per dataset in the supplementary information) |
| Normalization | recon-all default pipeline |
| Normalization template | *Describe the template used for normalization/transformation, specifying subject space or group standardized space (e.g. original Talairach, MNI305, ICBM152) OR indicate that the data were not normalized.* |
| Noise and artifact removal | Parcellations of cortical grey matter regions were visually inspected following the standardized ENIGMA quality control protocol (http://enigma.ini.usc.edu/protocols/imaging-protocols). Exclusions on the basis of this quality control resulted in the sample sizes used for the present study. |
| Volume censoring | *Define your software and/or method and criteria for volume censoring, and state the extent of such censoring.* |

## Statistical modeling & inference

| | |
|---|---|
| Model type and settings | A linear mixed random effects model was used: asymmetry index = fixed(diagnosis + age + sex) + random(dataset).<br>AI and age were coded as continuous variables, and diagnosis, sex, and dataset as factor variables. |
| Effect(s) tested | asymmetry index = fixed(diagnosis + age + sex) + random(dataset)<br>where the main effect of diagnosis on structural brain asymmetry was the primary effect of interest.<br>For a bilateral pair of structural measures, the asymmetry index = (L-R)/(L+R) |

Specify type of analysis: ☐ Whole brain ☐ ROI-based ☒ Both

| | |
|---|---|
| Anatomical location(s) | automated labeling in FreeSurfer, Desikan-Killiany atlas (34 cortical regions per hemisphere), plus subcortical volumes |
| Statistic type for inference (See Eklund et al. 2016) | P value, separate model per asymmetry index tested |
| Correction | Significance was assessed based on the P-values for the effects of diagnosis on AIs. The False Discovery Rate (FDR) was estimated separately for the 35 cortical surface area AIs (i.e., 34 regional AIs and one hemispheric total AI) and the 35 cortical thickness AIs, and again for the seven subcortical structures plus lateral ventricles, each time with a FDR threshold of 0.05. |

## Models & analysis

| n/a | Involved in the study |
|---|---|
| ☒ | ☐ Functional and/or effective connectivity |
| ☒ | ☐ Graph analysis |
| ☐ | ☒ Multivariate modeling or predictive analysis |

Multivariate modeling and predictive analysis

AI = fixed(diagnosis + age + sex) + random(dataset).
AI and age were coded as continuous variables, and diagnosis, sex, and dataset as factor variables.
